# Supplementary material for: Adjusting PSC culture for neural organoid generation
Source: Stem Cell Reports. 2025 Dec 4;21(1):102724. doi: 10.1016/j.stemcr.2025.102724 (PMC12925955; doi:10.1016/j.stemcr.2025.102724)
Supplement: Document S1. Figures S1–S5 [file mmc1.pdf]

Stem Cell Reports, Volume *21*

Supplemental Information

Adjusting PSC culture for neural organoid generation

Magdalena A. Sutcliffe, Pia Jensen, Joycelyn Tan, Charles A.J. Morris, Daniel J. Fazakerley, Martin R. Larsen, and Madeline A. Lancaster

Supplemental Materials

Supplemental Figures

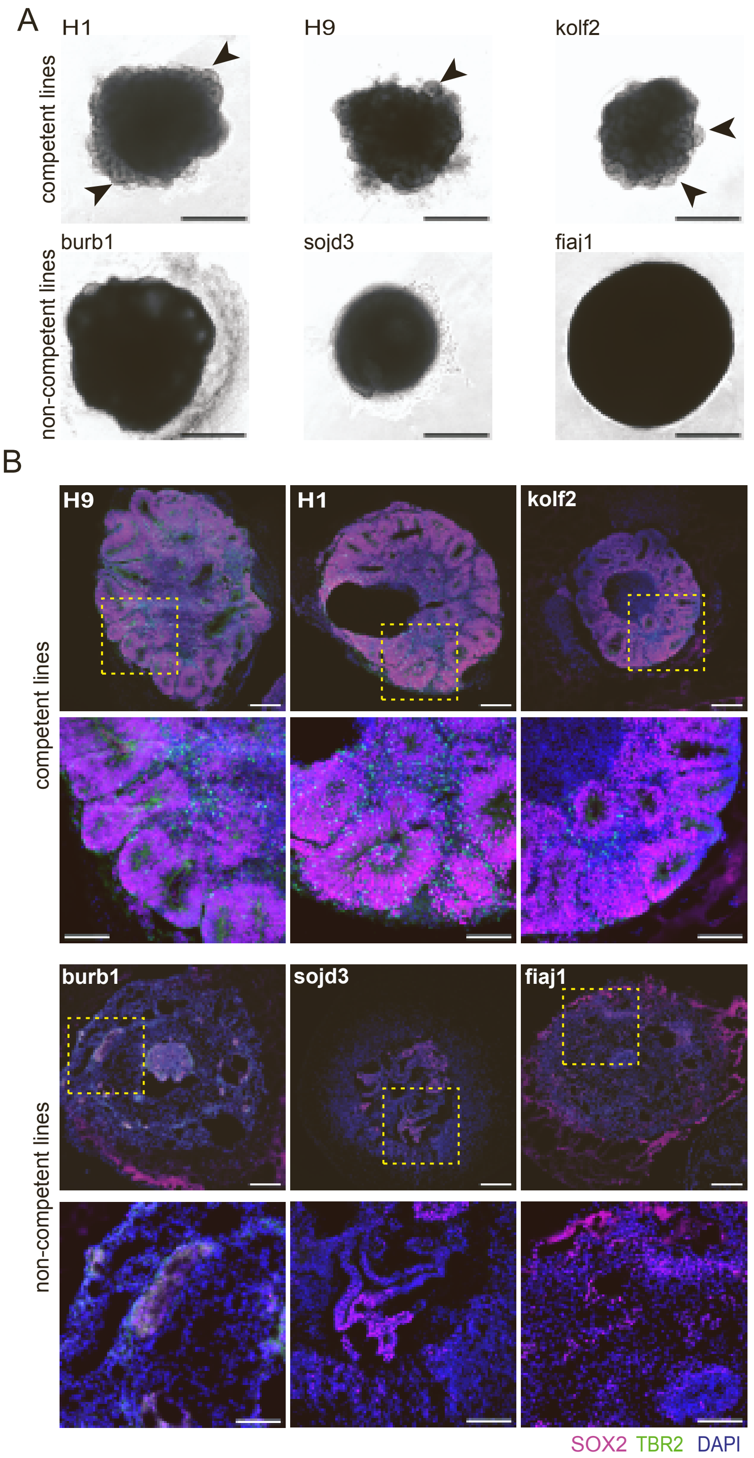

Figure S1. Morphology of organoids from competent and non-competent lines, related to Figure 1

A – representative bright field images of day 10 organoids, arrows indicate neuroepithelial buds, scale bars 500µm, B - representative images of day 20 organoids made from a competent and non-competent lines, scale bar 200 µm (upper panel) or 100 µm (bottom panel) for each line

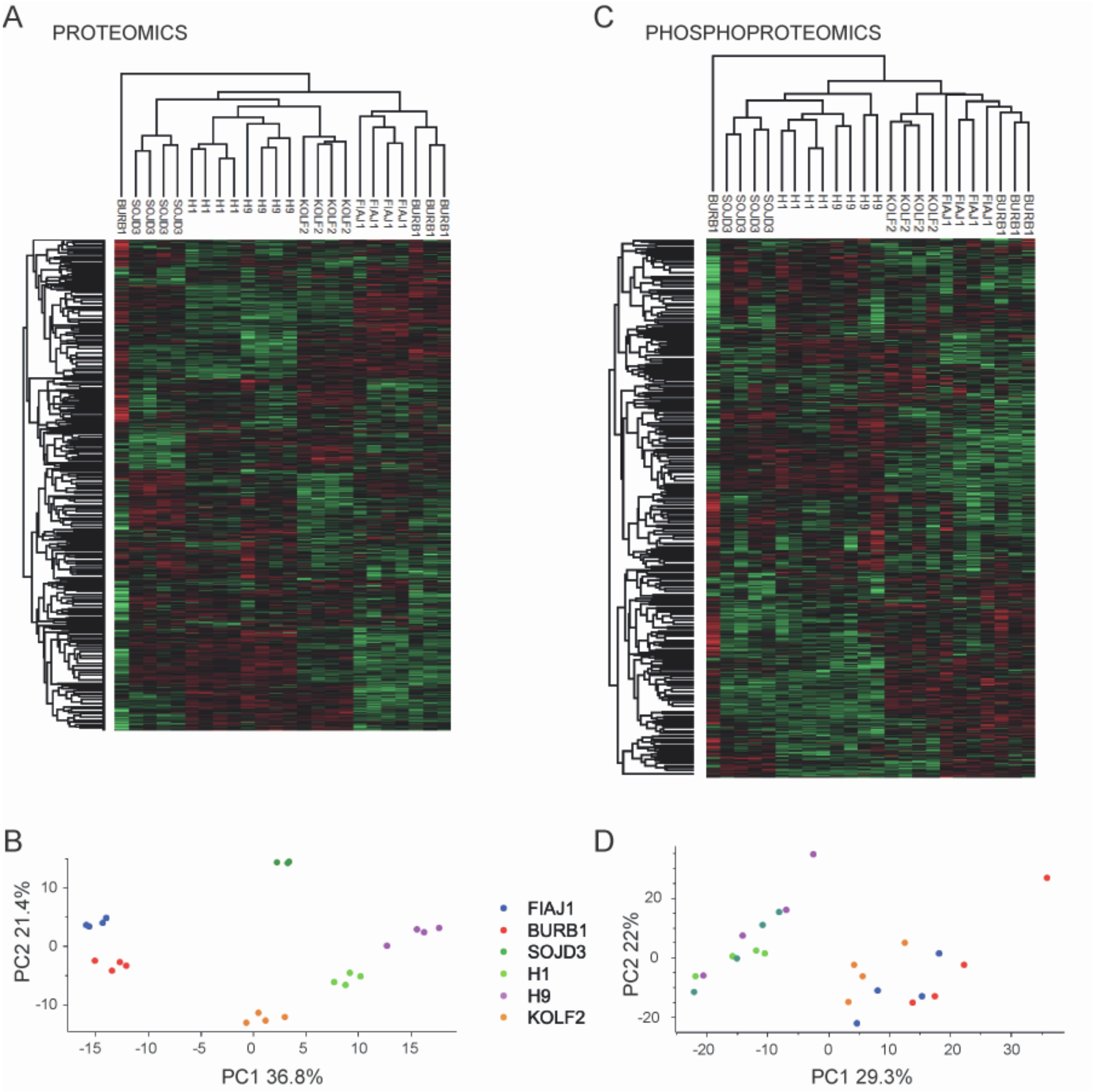

<u>Fig S2 Proteomic sample characterisation</u>

A – principal component analysis based on 5,798 non-modified proteins detected, plot shows PC1 vs PC2, B - heat map of the relative expression levels of the non -modified proteins, C - principal component analysis based on 12,143 phospho-peptides detected, plot shows PC1 vs PC2, D - heat map of the relative expression levels of phosphopeptides

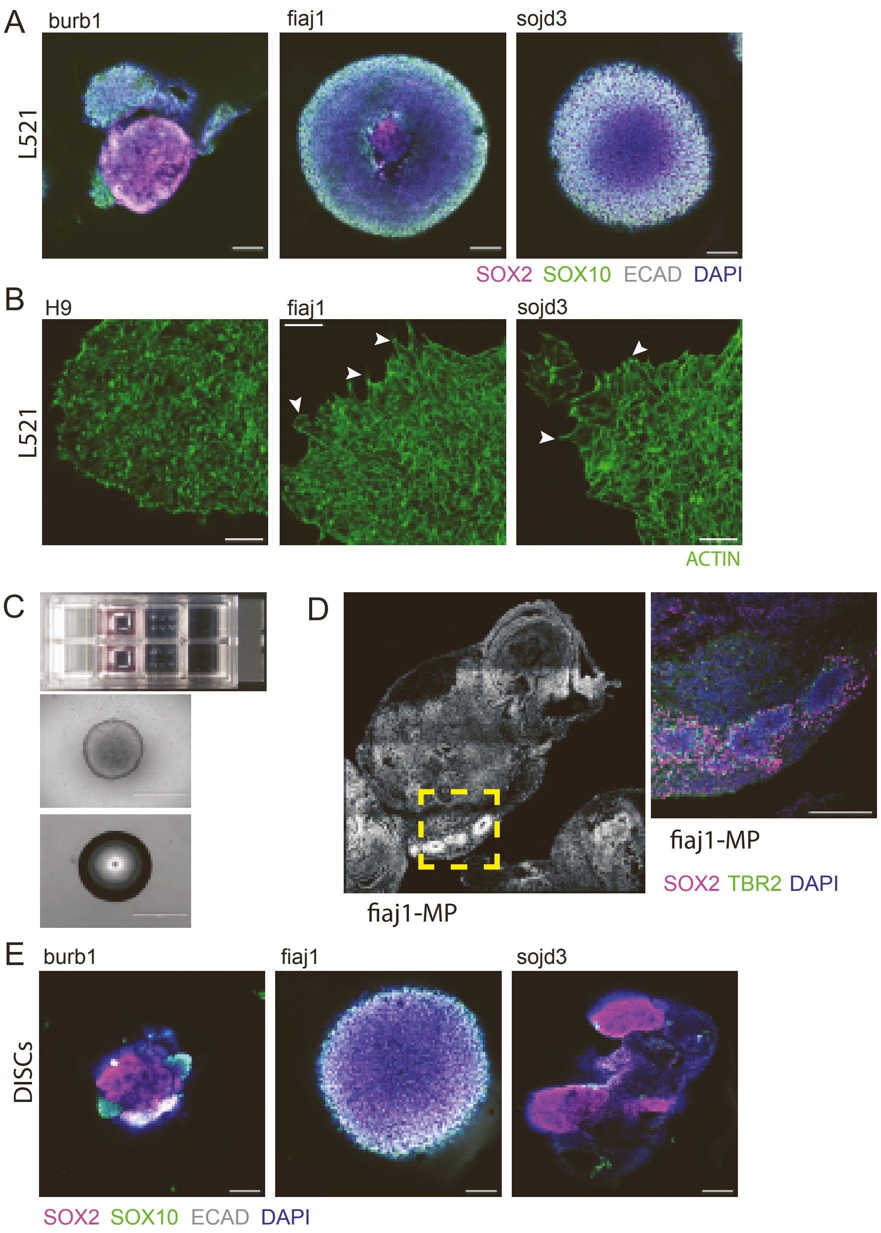

<u>Fig S3 Differences in response to treatments in non-competent lines, related to Figure 3</u>

A - day 10 organoids grown from cells cultured on L521, scale bars 200µm, B – preparation of micropattern colonies – droplets of L521 on a 8-well imaging slide, a droplet of L521 under phase contrast, and an attached micropatterned colony, scale bars 1000µm, C – overview and area of day20 fiaj1 organoids with morphology typical of a competent line, scale bar 200µm, D – day 10 organoids grown from cells cultured on L521 with FGF2 DISCs, scale bars 200µm

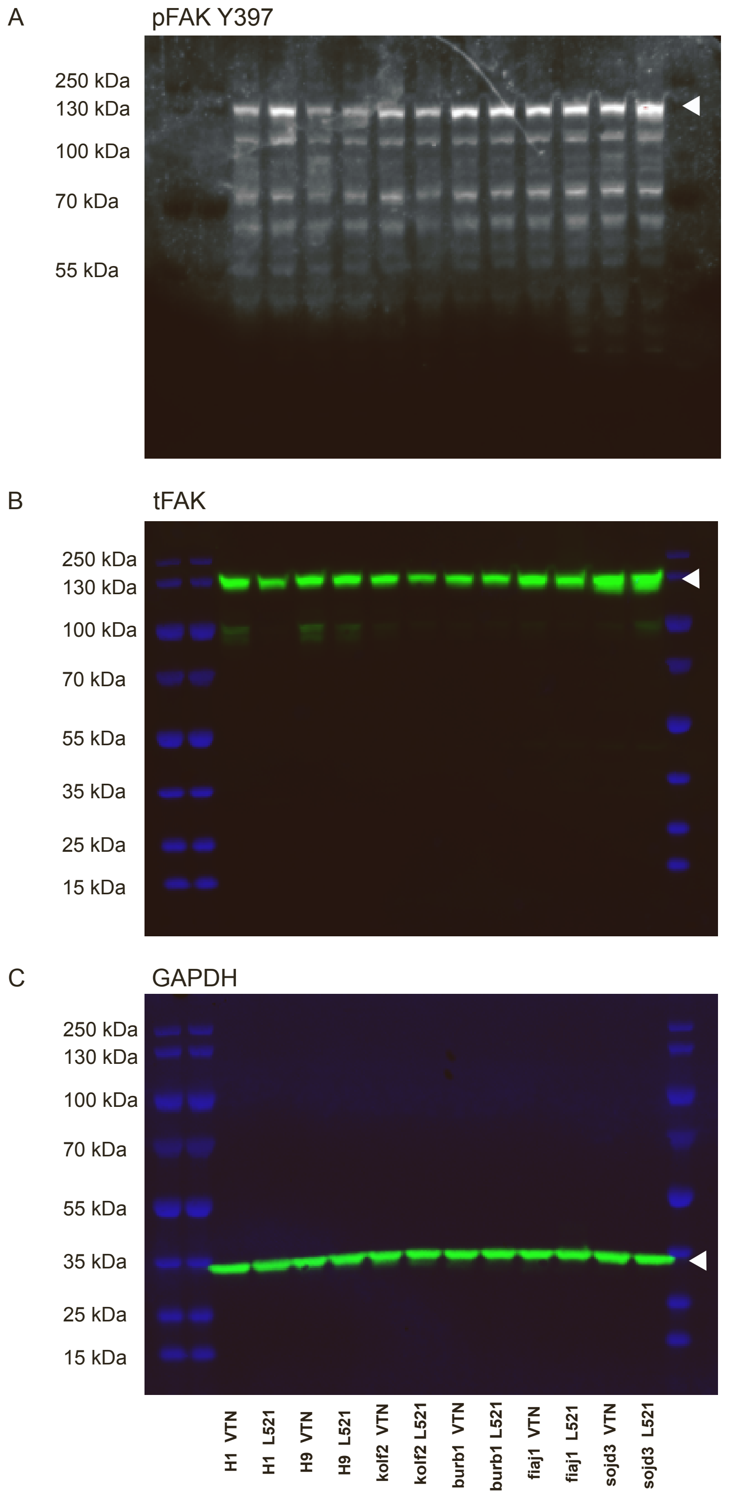

Fig S4 Full size unedited Western blot scans, relating to Figure 3

A – FAK phospho Y397, B – total FAK, C – GAPDH, white arrow heads show the correct band

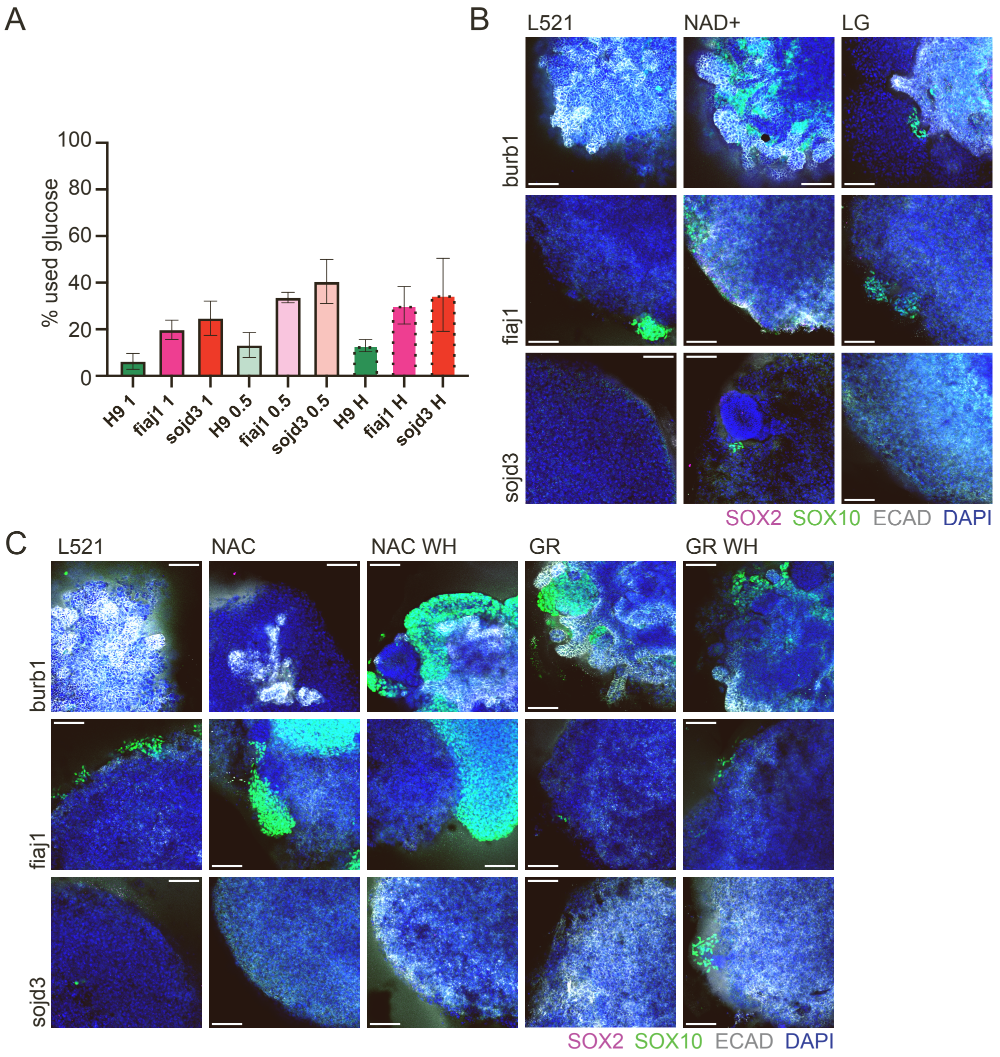

Fig S5 Manipulation of metabolic differences in non-competent lines, relating to Figure 4

A - glucose consumption after 24h, refers to Fig 4E, B – representative images of day 10 organoids made from cultures in E8 on L521, in low glucose medium or E8 supplemented with NAD+, scale bars 100µm, refers to Fig 4F, C - day10 organoids made from cultures with combinations of (GR) reduced glutathione and (NAC) N-acetyl cysteine and WH-4-023 (SRC inhibitor), lines represent means, bars 100µm, refers to Fig 4G

Supplemental Table Legends

All tables in separate .xlsx files

Supplemental Table S1. Quantification of images of day 20 organoids, related to Figure 1

Supplemental Table S2. Abundance of peptides in all samples (proteomics), related to Figure 1.

Supplemental Table S3. Abundance of peptides in all samples (phosphoproteomics), related to Figure 1.

Supplemental Table S4. Differentially abundant peptides, related to Figure 1. List of statistically differentially abundant peptides in proteomics data (Sheet1), in bold hits with fold change >1.5 and full results (Sheet2), condition 1 competent lines (H1, H9, kolf2), condition 2 non-competent lines (burb1, fiaj1, sojd3). List of statistically differentially abundant peptides in phosphoproteomics data (Sheet3), in bold hits with fold change >1.5 and full results (Sheet4), condition 1 competent lines (H1, H9, kolf2), condition 2 non-competent lines (burb1, fiaj1, sojd3).

Supplemental Table S5. Gene Ontology analysis of differentially abundant peptides, related to Figure 1. Data analysed using online tool pantherdb.org against all Homo sapiens genes, Fischer test with FDR correction. Sheet1 – proteomics upregulated in non-competent, Biological Process, Sheet2 - proteomics downregulated in non-competent, Biological Process, Sheet3 – phosphoproteomics upregulated in non-competent, Sheet4 - phosphoproteomics downregulated in non-competent

Supplemental Table S6. Variance Sensitive Fuzzy Clustering (VSClust) of the proteome data, relates to Figure 2.

Supplemental Table S7. Variance Sensitive Fuzzy Clustering (VSClust) of the phosphoproteome data, relates to Figure 2.

Supplemental Table S8. Gene Ontology analysis of differentially abundant peptides identified through Variance Sensitive Fuzzy Clustering (VSClust), relates to Figure 2. Sheet 1 – Cluster 2 unmodified peptides less abundant in non-competent, enriched GO Biological Process terms, Sheet 2 – Cluster 3 unmodified peptides more abundant in non-competent, enriched GO Biological Process terms, Sheet 3 – Cluster 3 unmodified peptides more abundant in non-competent, enriched GO Molecular Function terms, Sheet 4 - Cluster 4 phosphopeptides more abundant in non-competent, enriched GO Biological Process terms, Sheet 5 - Cluster 4 phosphopeptides more abundant in non-competent, enriched GO Molecular Function terms,

Supplemental experimental procedures

Cell lines

The ESC lines used in this study were H9 (WA09, female) and H1 (WA01, male) and were purchased from WiCell. The iPSC lines: burb1 (HPSI0714i-burb_1, hPSCreg WTSIi257-A, male), fiaj1 (HPSI0514i-fiaj_1, hPSCreg WTSIi301-A male), kolf2 (HPSI0114i-kolf_2, hPSCreg WTSIi018-B, male) and sojd3 (HPSI0314i-sojd_3, hPSCreg WTSIi073-A, female) were obtained from hispci.org. The master banks of iPSC lines were verified to be pluripotent in Pluritest and karyotyped by the vendor. The use of human ESCs used for this project was approved by the U.K. Stem Cell Bank Steering Committee and iPSCs and ESCs were approved by an ERC ethics committee and are registered on the Human Pluripotent StemCell Registry (hpscreg.eu).

Cell culture

All lines were grown from working cell banks of less than passage 50 and were cultured for less than 25 subsequent passages. All cell lines were cultured in Essential 8 (E8) medium (Thermo Fisher Scientific, A1517001) on TC-treated 6 well plates (Corning, 3516) coated with rh-VTN (Thermo Fischer Scientific, A14700) at 10 µg/ well. Cultures were split as clumps twice a week using 0.5mM EDTA at the ration of 1:4 to 1:10. When needed, cryopreservation was performed using mFreSR™ (StemCell Technologies, 05855). All cells were cultured without antibiotics or antimycotics to allow visualization of any potential contamination and if observed, cells were immediately discarded. Cells were routinely tested for mycoplasma using MycoAlert™ mycoplasma detection kit (Lonza, LT07). Where specified, culture plates were coated with rhL521 (Thermo Fisher Scientific, A29249) at 5 µg per well in DPBS with calcium and magnesium. For some experiments, cells were cultured in low glucose/low insulin medium, consisting of DMEM no glucose no pyruvate (Thermo Fisher Scientific, A1443001), Sodium bicarbonate 7.5%, L-Ascorbic acid 2-phosphate sesquimagnesium salt hydrate (Merck, A8960-5G), B27-A (Thermo Fisher Scientific, 17504044), N2 (Thermo Fisher Scientific, 17502001), MEMNEAA (Thermo Fisher Scientific, 11140050), Glucose 5mM, and growth factors FGF2 (100ng/mL, Peprotech, 100-18B-50UG) and TGFb (2 µg/mL Gibco, 100-21-10UG). This modified medium did not contain pyruvate, contained antioxidants from B27-A (DL Alpha Tocopherol Acetate, DL Alpha-Tocopherol, reduced glutathione, catalase and superoxide dismutase) and contained lower concentrations of insulin (5.3mg/L vs 19.4mg/mL) and glucose (5mM vs 17.5mM) than E8 medium. Geometrically constrained surfaces for cell attachment on glass slides (Ibidi, 80807) were prepared by applying droplets of L521 mixed 1:1 with DPBS containing calcium and magnesium (Gibco, 14040117). Gentle pipetting of a 0.5 µL portion of this solution produced a droplet of approximately 1 mm in diameter, forming an adhesive field for cells to attach (Fig S3 C). 250,000 single cells were seeded in each well with ROCK inhibitor (Y27632, Santa Cruz, sc-281642), allowed to attach for 2h and then washed to remove unattached cells.

Cerebral organoid generation

Cerebral organoids with telencephalic identity cells were generated using STEMdiff Cerebral Organoid Kit (StemCell Technologies, 08570), Fig 1A) according to manufacturer's protocol. Briefly, cultures at <80% confluence were washed with PBS and dissociated using Accutase (Gibco, A1110501). 9,000 cells were seeded in each well of a round bottom ultra-low adhesion 96 well plate (Corning) in EB medium with 10 µM Y27632 and left for 3 days to form embryoid bodies. Medium was replaced on day 3 with fresh EB medium without Y27632 and then changed to NI medium on day 5. EBs were embedded in Matrigel (Corning, 356235) droplets on day 7 and transferred to Expansion medium. On day 10 droplets were transferred to Maturation Medium. On day 13 Matrigel was removed using mechanical Dissociation and/or 30 minute incubation with Cell Recovery Solution (Corning, 354253) at 4°C, then tissues were transferred to fresh Maturation Medium and cultured with agitation.

Histological and immunohistochemical analysis

Samples were fixed in 4% PFA either overnight at 4 °C or at room temperature for 1h, then washed twice in PBS for 10 min. Samples for cryosectioning were incubated overnight in 30% sucrose in 0.2M PB (21.8 g/L Na_2HPO_4, 6.4 g/L NaH_2PO_4 in dH_2O), embedded in gelatine (7.5% gelatine, 10% sucrose in 0.2 M PB), and plunge frozen in 2-methylbutane (Sigma-Aldrich, M32631) at below -30 °C. Frozen blocks were sectioned at the thickness of 20 µm and stained. Wholemount samples were stained for 24-48h with primary antibodies followed by 24-48 h with secondary antibodies in a buffer of 4% normal donkey serum and 0.25% Triton-X-100 in PBS.

Antibodies and stains

Primary antibodies used in this study were as follows: SOX2 (Abcam, ab97959, 1:200 for IF), TBR2 (R&D Systems, AF6166 1:200 for IF), HuC/D (Invitrogen, A2127, 1:200 for IF), FAK (Abcam, ab40794, 1:1000 for WB), pFAKY397 (Thermo Fisher Scientific, 44-624G, 1:200 for IF, 1:1000 for WB), GAPDH (Abcam, ab8245, 1:5000 for WB), SOX10 (R&D Systems, AF2864, 1:100-1:200 for IF), ECAD (BD Transduction, 610181, 1:400 for IF), TOM20 (Santa Cruz, sc-17764 1:500 for IF). Secondary antibodies used were: Donkey a-Ms 488 (Life Technologies, A21202), Donkey a-Rb 488 (Life Technologies, A21206), Donkey a-Sh 488 (Life Technologies, A11015), Donkey a-Ms 568 (Life Technologies, A10037), Donkey a-Rb 568 (Life Technologies, A10042), Donkey a-Ms 647 (Life Technologies, A31571), Donkey a-Rb 647 (Life Technologies, A31573), Donkey a-Sh 647 (Life Technologies, A21448). All secondary antibodies were used at 1:500 dilution. Nuclei were counterstained with 0.1 µg/mL DAPI (Merck, 268298). F-actin was detected with ActinGreen 488 ReadyProbes Reagent (Alexa fluor 488 conjugated phalloidin, Invitrogen).

Imaging and image analysis

Images were acquired on Zeiss LSM 710 or Zeiss LSM 780 systems with each channel as separate track. Images were acquired at x100, x200 or x630 magnification. Raw images were processed using FIJI and brightness and/or contrast were adjusted where needed for clarity.
Actin structure quantification: Prior to image analysis all images were deconvolved with Huygens Professional Medium version 24.10.0p0 (Scientific Volume Imaging, The Netherlands, http://svi.nl), using the CMLE algorithm, with following parameters -it 30 -q 0.01 -snr 19.73 -acuity 0.00 -acuityMode on -bg 0.0 -bgMode lowest -bgRadius 0.7 -pad auto -mode fast -reduceMode auto -blMode off -brMode auto -varPsf off -tclReturn. Deconvolved images were processed in FIJI Version 2.16.0/1.54p using top hat filter with radius 4, then threshold default dark to create a binary mask. The “analyse particles” function was applied with a minimum size of 0.10 and the major and minor axis parameters were saved as length and width, respectively.
Quantification of pFAK immunofluorescence: Images were deconvolved as described above and processed in FIJI Version 2.16.0/1.54p. Areas where cells were present in an image were detected based on the DAPI channel using Image>Adjust>Threshold with Default settings, adjusted manually and saved as DAPI ROI. Within DAPI ROI fluorescent signal was isolated using Image>Adjust>Threshold with Default settings and the % of total area was calculated as (signal area)/(DAPI ROI area).
Quantification of organoid morphology: Images were processed in FIJI Version 2.16.0/1.54p. A single image per organoid was quantified. Total area of organoid optical section was detected based on the DAPI channel using Image>Adjust>Threshold with Default settings, adjusted manually and saved as DAPI ROI. Within DAPI ROI fluorescent signal was isolated using Image>Adjust>Threshold with Default settings and the % of total area was calculated as (signal area)/(DAPI ROI area).
Quantification of mitochondria morphology: Prior to image analysis all images were deconvolved with Huygens Professional Medium version 24.10.0p5 (Scientific Volume Imaging, The Netherlands, http://svi.nl), using the CMLE algorithm, with following parameters -it 20 -q 0.01 -snr 19.86 -acuity 0.00 -acuityMode on -bg 0.0 -bgMode lowest -bgRadius 0.7 -pad auto -mode fast -reduceMode auto -blMode off -brMode auto -varPsf off -tclReturn. Mitochondria were segmented using Huygens Object Analyzer function with the following parameters: -gaussMode 0, -threshAbs 13.5, -seed 37.65, -

garbage 100, -segMode watershed, -fragmentation 100, -seedMode sparse and small particles geometry report was generated with axial roundness and rough roundness values used for analysis.

Statistical analysis

Data were collected across two independent batches of organoids or cells. The number of samples, "n" was 6 for all the experiments except for protemics. For cell experiments "n" represents an area analysed and all measurements of individual structures were averaged per area. For organoid experiments "n" represents individual organoids. for Data were analysed in Prism version Version 10.4.1 and 10.4.2. Where comparisons were made between competent and non-competent cells, a Mann-Whitney test was applied and the values analysed were the means in each group. Where comparisons were made between different treatments, and in glucose to lactate conversion experiments, a Kruskal-Wallis test was used. Data are presented as individual datapoints with lines representing mean+/-SD, except from figures 4E and S4A where data are presented as columns representing mean and error bars representing SD.

Protein extraction, digestion and Tandem Mass Tag labeling

At the point of organoid generation, 60% of Accutase dissociated cells were pelleted at 200rcf, the supernatant was removed, and the remaining pellets were frozen at -70°C. Material was collected from 4 independent organoid batches for each cell line. Cell pellets were dissolved in 150 µL lysis buffer consisting of 1% (w/v) sodium deoxycholate (SDC, Sigma), 10 mM Dithiothreitol (DTT, Sigma), and 50 mM triethylammonium bicarbonate (TEAB, Sigma) at pH 8 and sonicated on ice 4x for 10s using a probe sonicator at 40% amplitude. Protein concentrations were measured using a NanoPhotometer® N60 (Implen) against a standard curve of HeLa cell protein extract. 50 µg of each sample was alkylated with 20 mM Iodoacetamide (IAA, Sigma) for 30 minutes in the dark followed by digestion with 5% (w/w) in-house methylated trypsin (Sigma)(Heissel et al., 2018) for 4h at 37°C.
Thereafter the peptide samples were labelled with Tandem Mass Tag (TMT) 16plex Isobaric label Reagents (Thermo Scientific) according to the manufacturer's instructions.
Two sets of a TMTpro 16-plex were used, in each TMT set one channel was used for labelling of a pooled sample to enable comparison of samples between the TMT sets. The labelling reaction was checked by LC-MS/MS analysis to ensure proper labelling of all TMT channels, excess reagent was quenched using 5% hydroxylamine (v/v) (Thermo Scientific) for 15 min at RT. After incubation, the labelled peptides were mixed 1:1 and the pooled TMT samples were acidified with 2% Formic acid (FA) and vortexed to pellet SDC. The samples were centrifuged at 20,000 g for 15 min at RT and the supernatant was transferred to a new tube and dried by vacuum centrifugation.

Enrichment of phosphorylated peptides

The TMT labelled peptide mixture was dissolved in a solution of 80% acetonitrile (ACN), 5% trifluoroacetic acid (TFA), and 1 M glycolic acid (Sigma), and incubated with 0.6 mg TiO_2 beads (Titansphere 10 µm, GL Sciences) per 100 µg peptide for 15 min at RT with vigorous shaking to enrich the phosphorylated peptides. The beads were centrifuged briefly, and the supernatant transferred to a new tube with 0.3 mg TiO_2 beads per 100 µg peptide. After 10 min incubation at RT with vigorous shaking and a brief centrifugation the supernatant was collected. The beads were subsequently washed with 80% ACN/1% TFA and 10% ACN/0.1% TFA. The supernatant with the unbound TiO_2 fraction and the washing fractions, both containing the non-modified peptides, were combined. The phosphorylated peptides were eluted from the beads by incubation with 1.5% ammonium hydroxide solution (Sigma) at RT and pH 11.3with vigorous shaking. The beads were spun down and the supernatant was passed through C8 material from a 3M Empore^{TM} disk (Sigma). Any remaining peptides were eluted from the disk with 30% ACN and all peptide samples were dried. Since sialylated glycopeptides also bind to the TiO_2 beads(Larsen et al., 2007), the sample was deglycosylated with N-glycosidase F (Biolabs) and Sialidase A (Prozyme) in 50 mM TEAB, pH7.5 at 37°C ON.

High-pH fractionation

To reduce the complexity of the samples, non-modified and phosphophospho-peptides were fractionated by High-pH chromatography prior to nanoLC-MS/MS analysis.
The peptide samples were dissolved in 30 µL solvent A (20 mM ammonium formate, pH 9.5) and loaded onto an Acquity UPLC™M-Class CSH™C18 column (Waters) using a Dionex Ultimate 3000 HPLC system (Thermo Scientific). Approximately 100 µg of the non-modified peptide samples was fractionated, whereas the whole of the phosphopeptide samples were fractionated.
Separation of the peptides was performed using a 70-minute gradient from 2 to 95% solvent B (80% ACN, 20% solvent A) in solvent A, at a flow rate of 0.1 µL/min. The fractions were collected every 60 seconds into a final of 12 concatenated fractions in a 96-well plate (Axygen), dried by vacuum centrifugation, and stored at -20°C.

Reversed-phase nanoLC-ESI-MS/MS

Each high pH fraction was resuspended by adding 3 µL of solvent A (0.1% FA) and loaded in a volume of 2.5 µL onto an analytical column on an EASY-nLC 1000 system (Thermo Scientific).
The analytical column was a 21 cm long fused silica capillary (75 µm inner diameter) and packed with ReproSil-Pur C18 AQ 1.9 µm reversed-phase material (both resins Dr. Maisch Ammerbuch-Entringen). The peptides are eluted with an increasing concentration of organic solvent (solvent B: 95% ACN, 0.1% FA) over a gradient of 120 min in the following manner: from 2% to 25% solvent B in 100 min, 25-40% in 20 min and 40-95% in 1 min. The flow was 300 nL/min. The nLC was online connected to an Orbitrap Eclipse™ Tribrid™ mass spectrometer (Thermo Scientific) operated at positive ion mode with data-dependent acquisition. The Orbitrap acquired the full MS scan with an automatic gain control (AGC) target value of 300% ($3x10^6$) ions and a maximum injection time of 50 ms. Each MS scan is acquired at high-resolution (120,000 full width half maximum (FWHM)) at m/z 200 in the Orbitrap, with a mass range of 350-1600 Da. For the non-modified peptides, the peptide fragmentation was performed using the SPS-MS3 method with real time database searching(Schweppe et al., 2020). Briefly, each peptide was selected (0.7Da window) and fragmented in the linear ion-trap using CID with a normalized collision energy of 35% and an activation time of 10 ms. Each MSMS spectrum was subjected to a brief 20-30 ms database search against a Human uniprot reviewed database using a database search program built into the MS computer. If the resulting MSMS database search received a confident match in the database, the same ion was reselected and fragmented in the linear ion-trap. Subsequently, the 10 most intense fragment ions originating from the identified peptide were reselected, fragmented using HCD fragmentation (NCE 55), and scanned out in the orbitrap with 30.000 in resolution optimised for resolving of the TMT reporter ions. The eclipse workflow was set to automatic calculation of number of peaks that could be selected within a 3 second duty cycle. For the phosphopeptides, the fragmentation was performed using HCD NCE 36, with the following settings for ion detection; resolution 50K FWHM, maximum injection time 200 ms, and AGC target 200%. The MSMS was performed with a cycle time of 3 sec. All raw data were viewed in Thermo Xcalibur v3.0.

Mass spectrometry data analysis

The raw data were processed using Proteome Discoverer (v2.5, ThermoFisher, PD2.5) and all data were cross referenced against a Human Uniport Reviewed database. The non-modified peptides were searched for using the SEQUEST HT search algorithm only, while the phosphopeptide data were searched for initially using an in-house Mascot search algorithm and then by the SEQUEST HT search algorithm. The data were searched with 10 ppm accuracy in MS and 0.8 Da in MSMS mode (linear ion trap MSMS) for the non-modified peptides, and 0.05 Da for the phospho-peptides. The quantitation was performed using the MS3 HCD spectra for the non-modified peptides, and MS2 HCD spectra for the phospho-peptides. Database searches were performed with the following parameters: TMTpro 16-plex (Lys and N-terminal) as fixed modifications and a maximum of 2 missed cleavages for trypsin. Additionally, for the phospho-peptides, the search was performed with

phosphorylation of serine/threonine/tyrosine (S/T/Y) and deamidation of asparagine (N) as variable modifications. All identified peptides were filtered against a Decoy database using Percolator with a false discovery rate (FDR) of 0.01 (FDR < 0.01). Only peptides with rank 1 were considered for further analysis. Only proteins with more than 1 unique peptide were considered for further analysis in the non-modified group.

Quantitative analysis was based on 4 biological replicates. Quantification across the 2 sets of TMTpro 16-plex was normalized based on a common reference channel containing a mix of all samples. The relative abundances of the non-modified and phosphopeptides were normalized using PD2.5.

Principal component analysis was performed in PD2.5 and Perseus v1.5.4.1 to evaluate the separation between the replicates and the sample groups. Heatmaps were prepared in Perseus v1.5.4.1 with k-means clustering and Euclidean distance. Statistical testing to identify significant differences between the noncompetent and the competent cell lines was performed using PolyStest (http://computproteomics.bmb.sdu.dk:443/app_direct/PolySTest)(Schwämmle et al., 2020) applying the Limma test with FDR<0.05. Results were evaluated using Panther (https://pantherdb.org) against the background of all human genes with FDR<0.01 cut-off to identify enriched GO annotations. Further Cluster analysis was performed using Variance sensitive fuzzy clustering (VSClust) (http://computproteomics.bmb.sdu.dk:8192/app/VSClust)(Schwämmle & Jensen, 2018). Results from the cluster analysis were further evaluated using Panther against the background of all detected peptides with FDR<0.01 cut-off. Enrichment bar plots were generated using a custom python script.

Immunoblotting

Cells on culture plates were washed twice in ice-cold PBS and lysed with modified RIPA buffer (mRIPA: 1% Triton-X, 0.1% SDS, 150 mM NaCl, 50 mM Tris pH 7.4, 2 mM EDTA, 12 mM sodium deoxycholate) freshly supplemented immediately with protease (Thermo Fisher, 78430) and phosphatase (Sigma-Aldrich, 4906845001) inhibitors. The protein concentration of the samples was measured using the Quick Start Bradford Dye Reagent (Bio-Rad, 5000205). 10 µg of total protein per sample was resolved by SDS-PAGE (4-20% gels) and transferred to Amersham Hybond P 0.45 PVDF blotting membranes (GE Healthcare, 10600023). Membranes were blocked overnight at 4°C in 5% skim milk powder or in 5% BSA and 0.1% Tween in PBS for working with phospho-specific antibodies. They were then incubated with primary antibodies overnight at 4 °C in 5% skim milk powder or in 5% BSA and 0.1% Tween in PBS. HRP-conjugated goat anti-rabbit (Dako, P0448, 1:3000), Dylight800 4xPEG conjugated anti-mouse (Cell Signalling Technology 5257P, 1:3000) or Dylight800 4xPEG conjugated anti-rabbit (Cell Signalling Technology 5151P, 1:3000) secondary antibodies were incubated for 1 hr at room temperature. The blots were developed with ECL Prime enhanced chemiluminescent detection reagent (GE Healthcare, RPN2232) and imaged using a Chemidoc MP system (BioRad), or imaged directly on the same system. Images were quantified in FIJI Version 2.16.0/1.54p and the final results are presented as a ratio of pFAK/FAK signal.

Supplemental references

Heissel, S., Bunkenborg, J., Kristiansen, M. P., Holmbjerg, A. F., Grimstrup, M., Mørtz, E., Kofoed, T., & Højrup, P. (2018). Evaluation of spectral libraries and sample preparation for DIA-LC-MS analysis of host cell proteins: A case study of a bacterially expressed recombinant biopharmaceutical protein. *Protein Expression and Purification*, *147*, 69–77. https://doi.org/10.1016/j.pep.2018.03.002

Larsen, M. R., Jensen, S. S., Jakobsen, L. A., & Heegaard, N. H. H. (2007). Exploring the Sialiome Using Titanium Dioxide Chromatography and Mass Spectrometry. *Molecular & Cellular Proteomics*, *6*(10), 1778–1787. https://doi.org/10.1074/mcp.M700086-MCP200

Schwämmle, V., Hagensen, C. E., Rogowska-Wrzesinska, A., & Jensen, O. N. (2020). PolySTest: Robust Statistical Testing of Proteomics Data with Missing Values Improves Detection of Biologically Relevant Features. *Molecular & Cellular Proteomics*, *19*(8), 1396–1408. https://doi.org/10.1074/mcp.RA119.001777

Schwämmle, V., & Jensen, O. N. (2018). VSClust: Feature-based variance-sensitive clustering of omics data. *Bioinformatics*, *34*(17), 2965–2972. https://doi.org/10.1093/bioinformatics/bty224

Schweppe, D. K., Eng, J. K., Yu, Q., Bailey, D., Rad, R., Navarrete-Perea, J., Huttlin, E. L., Erickson, B. K., Paulo, J. A., & Gygi, S. P. (2020). Full-Featured, Real-Time Database Searching Platform Enables Fast and Accurate Multiplexed Quantitative Proteomics. *Journal of Proteome Research*, *19*(5), 2026–2034. https://doi.org/10.1021/acs.jproteome.9b00860
