# Supplementary material for: Adjusting PSC culture for neural organoid generation
Source: Stem Cell Reports. 2025 Dec 4;21(1):102724. doi: 10.1016/j.stemcr.2025.102724 (PMC12925955; doi:10.1016/j.stemcr.2025.102724)
Supplement: Document S2. Article plus supplemental information [file mmc10.pdf]

STEM CELL REPORTS ISSCR
Article
OPEN ACCESS

Adjusting PSC culture for neural organoid generation

Magdalena A. Sutcliffe,[1] Pia Jensen,[3] Joycelyn Tan,[4] Charles A.J. Morris,[1] Daniel J. Fazakerley,[4] Martin R. Larsen,[3] and Madeline A. Lancaster[1,2,5,*]
[1]MRC Laboratory of Molecular Biology, Cambridge, UK
[2]Cambridge Stem Cell Institute, University of Cambridge, Cambridge, UK
[3]Protein Research Group, University of Southern Denmark, Odense, Denmark
[4]Metabolic Research Laboratories, Institute of Metabolic Science, University of Cambridge, Cambridge, UK
[5]Lead contact
*Correspondence: madeline.lancaster@mrc-lmb.cam.ac.uk
https://doi.org/10.1016/j.stemcr.2025.102724

SUMMARY

Cerebral organoids generated according to unguided protocols produce neural tissue with exceptional cell diversity and fidelity to *in vivo*. However, with only minimal extrinsic intervention, the importance of high-quality starting material becomes paramount. Understanding quality and how to maintain it throughout prolonged culture is therefore a crucial foundation for successful organoid differentiation. In this study, we investigate the proteome and phosphoproteome of human pluripotent stem cells to uncover the mechanisms that drive neural organoid competence. We identify aberrant cell-extracellular matrix interaction and increased oxidative metabolism as hallmarks of poor neural differentiators. Drawing on the proteomic data and published literature, we test culture conditions with improved coating matrix, reduction of oxidative stress, and sustained fibroblast growth Factor 2 (FGF2) supply. These adjustments provide some improvement to differentiation, highlighting the importance of optimal culture conditions to maintain high-quality stem cells but also suggesting cell-intrinsic sources of variability.

INTRODUCTION

In the process of gastrulation, a mammalian embryo generates all major primordia that will later generate all tissue types of the fully formed body. This ability is retained in pluripotent stem cells (PSCs) cultured *in vitro*, either embryonic stem cells (ESCs) (Thomson et al., 1998) or induced pluripotent stem cells (iPSCs) (Takahashi and Yamanaka, 2006).

In vitro, iPSCs derived from mouse, human, and other primates have been used to generate a wide range of tissues and cell types, with insights from *in vivo* development guiding the selection of signaling molecules to direct lineage specification.

In the absence of patterning signals, ESCs and epiblast cells default to a neural fate upon pluripotency exit (Lippmann et al., 2014; Wataya et al., 2008; Ying et al., 2003), a feature recapitulated in cerebral organoids (Lancaster et al., 2013). These unguided organoids predominantly generate cortical identities alongside adjacent structures such as the hem, choroid plexus, and retina, organized in a spatially and temporally relevant manner reminiscent of the developing human brain (Renner et al., 2017). By providing tissue context and faithfully mirroring natural development and physiology on a microscale, organoids serve as powerful models for studying human development and disease. Single-cell and omics techniques allowed benchmarking against *in vivo* tissues and validated organoids as tools for studying biological processes previously inaccessible due to technical or ethical limitations (Benito-Kwiecinski et al., 2021; Camp et al., 2015; Pollen et al., 2019; Velasco et al., 2019). However, the complexity and fidelity to *in vivo* come at a cost—the differentiation processes is sensitive to inter-cell line variability and the physiological state of the PSCs (Glass et al., 2024; Ideno et al., 2022; Lancaster and Knoblich, 2014; Sandoval et al., 2024; Watanabe et al., 2022).

Previous research has advanced our understanding of variability and differentiation bias among PSC lines and established guidelines for selecting the most appropriate lines for generating specific tissue types (Andrews et al., 2022; Bock et al., 2011; Kilpinen et al., 2017; Merkle et al., 2022; Pantazis et al., 2022; Puigdevall et al., 2023). More recently, a comprehensive analysis of the relationship between transcriptomic profiles and neural differentiation competency has provided an invaluable resource for identifying suitable lines and markers of good and poor neural differentiators (Jerber et al., 2021). However, the question whether and how differentiation competency can be modified remains open. While donor origin accounts for nearly half of the observed variability among iPSCs (Kilpinen et al., 2017), culture conditions represent a close second (Mirauta et al., 2020). In this study, we sought to manipulate cerebral organoid competency by optimizing and standardizing culturing conditions, guided by the proteome and phosphoproteome of successful and unsuccessful differentiators. We focused on the proteome as the most accurate, sensitive, and dynamic readout of cell state (Brenes et al., 2021). This approach provides a deeper understanding of the molecular mechanisms

© 2025 MRC Laboratory of Molecular Biology.
Published by Elsevier Inc. on behalf of International Society for Stem Cell Research.
This is an open access article under the CC BY license (http://creativecommons.org/licenses/by/4.0/).

involved and guides the refinement of culturing techniques, with the aim to standardize and improve organoid development.

RESULTS

Proteomic characterization of organoid competency

We selected a panel of six cell lines, comprising three organoid-competent lines (H9, H1, and kolf2) and three lines (sojd3, burb1, and fiaj1) previously demonstrated to be incompetent (Jerber et al., 2021). Each group included both male and female lines to account for differences due to sex chromosome makeup. All cell lines were cultured in identical conditions, namely in Essential 8 (E8) medium and on vitronectin (VTN)-coated plates to maintain a fully defined culture environment.

Each line was differentiated toward unguided cerebral organoids (Giandomenico et al., 2021; Lancaster et al., 2013; Lancaster and Knoblich, 2014). At the point of organoid generation, we saved 60% of the dissociated cells for proteomic analysis and used the remaining material to produce embryoid bodies (Figure 1A). We then monitored the development of the organoids to confirm their competent or non-competent phenotype, allowing us to link the organoid differentiation outcomes to each cell line.

Differences in organoid morphology were apparent as early as day 10. Competent cell lines produced organoids with well-defined neural buds, while non-competent lines formed dark structures lacking the surface features indicative of neural structures, such as inflection points, and instead occasionally developed less cell-dense areas that later became cysts (Figure S1A). Organoids exhibiting correct morphology at day 10 typically progressed to high-quality mature organoids, whereas those with poor initial morphology either failed to improve or deteriorated over time. This was consistent with our previous observations that early organoid morphology reliably predicts mature organoid quality (Chiaradia and Lancaster, 2020).

We assigned a final organoid quality score at day 20, when expression of the dorsal forebrain marker TBR2 was apparent (Figures 1B and S1B). Cell lines were classified as competent if they produced well-structured organoids with cortical buds comprising pseudostratified SOX2^{+} progenitors and a visible, scattered layer of dorsal forebrain-specific TBR2^{+} intermediate progenitors in at least some regions (Figure 1C). Expression of TTR2 (choroid plexus marker), DLX2 (ventral forebrain marker), FOXA2 (marker of floor plate), and VIM (mesenchymal cell maker) proved less informative for the distinction between successful and unsuccessful differentiations (Figure 1C). Based on the day 20 scores, we classified the starting stem cells as either competent or non-competent (Figure 1C; Table S1).

The starting cells saved at the embryoid body generation step were processed for quantitative proteomic analysis using tandem mass tag (TMT) liquid chromatography-tandem mass spectrometry (LC-MS/MS). This approach identified 7,792 proteins, of which 5,798 were identified with two or more unique peptides and were used for further analysis (Table S2). 13,697 of the 23,220 identified phospho-peptides were detected in all samples and were used for further analysis (Table S3). Dimensionality reduction was used to examine how the samples grouped together. The 4 biological repeats for each cell line clustered very closely together both for proteomics and phospho-proteomic data. As expected, proteomic profiles of the competent lines H9 and H1 clustered the closest but also near to kolf2. The non-competent lines fiaj1 and burb1 also clustered together, whereas sojd3 samples clustered separately (Figures S2A and S2B). Interestingly, in the phospho-proteomic analysis, sojd3 clustered with the competent lines H9, H1, and kolf2, whereas fiaj1 and burb1 clustered separately (Figures S2C and S2D).

Analysis of differentially abundant peptides in non-competent vs. competent lines revealed 129 upregulated and 78 downregulated peptides (fold change 1.5, Figure 1D; Table S4). Analysis of Gene Ontology (GO) biological processes (BP) for upregulated peptides returned terms related to metabolism, basement membrane, and cytoskeletal organization, whereas downregulated proteins were involved in NAD metabolism and white blood cell proliferation (Figures 1E and 1F; Table S5). Phospho-proteomic analysis indicated 136 upregulated and 63 downregulated peptides in non-competent lines (Figure 1G; Table S4). Upregulated proteins were involved in cell adhesion, chromatin organization, and transcription regulation, whereas downregulated proteins played roles in ribosome biogenesis (Figures 1H and 1I; Table S5).

Further analysis using variance-sensitive fuzzy clustering (Schwämmle and Jensen, 2018) of the proteome data yielded four clusters (Table S6). Cluster 2 represented proteins that were decreased in non-competent lines, while cluster 3 proteins were increased in non-competent lines (Figure 2A). We then analyzed hits from clusters 2 and 3 for enrichment of GO BP and molecular functions (MF). Cluster 2 showed only two processes enriched more than 2-fold (Figure 2B; Table S6) and no statistically significant results for GO MF. In contrast, cluster 3 revealed several terms enriched for the tricarboxylic acid (TCA) cycle, aerobic respiration, and the actin cytoskeleton in both BP and MF (Figures 2C and 2D; Table S6).

An analogous analysis of the phosphoproteome resulted in six clusters (Table S7). Cluster 5 hits were decreased in non-competent cells, while cluster 4 hits were increased (Figure 2E). Panther GO term analysis did not yield any

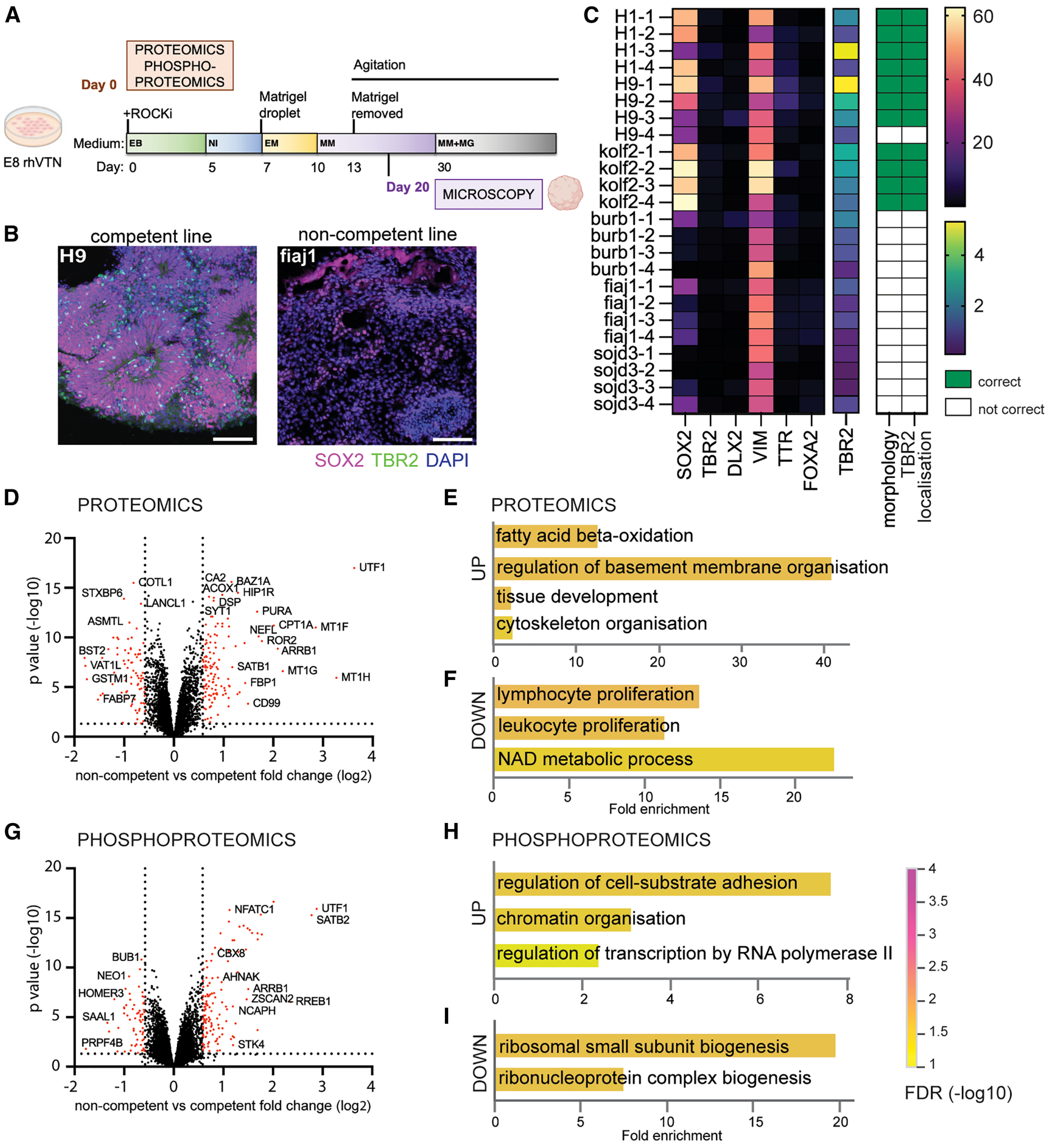

Figure 1. Proteomic analysis of competent and non-competent lines

(A) Schematic of the unguided brain organoid protocol used in this study with indicated sample collections for proteomics and phosphoproteomics, and microscopy.

(B) Representative images of organoids at day 20 made from a competent and non-competent line; scale bars, 100 μm.

(C) Heatmap of quantification of immunofluorescent markers used to assess cerebral organoid success.

(D) Volcano plot of differentially abundant (1.5 FC) peptides non-competent vs. competent, $n = 4$ independent batches, Limma test.

(E) Selected GO BP terms overrepresented in non-competent cells (proteome).

(F) Selected GO BP terms underrepresented in non-competent cells (proteome).

(G) Volcano plot of differentially abundant (1.5 FC) phosphopeptides non-competent vs. competent, $n = 4$ independent batches, Limma test.

(H) Selected GO BP terms overrepresented in non-competent cells (phosphoproteome).

(I) Selected GO BP terms underrepresented in non-competent cells (phosphoproteome).

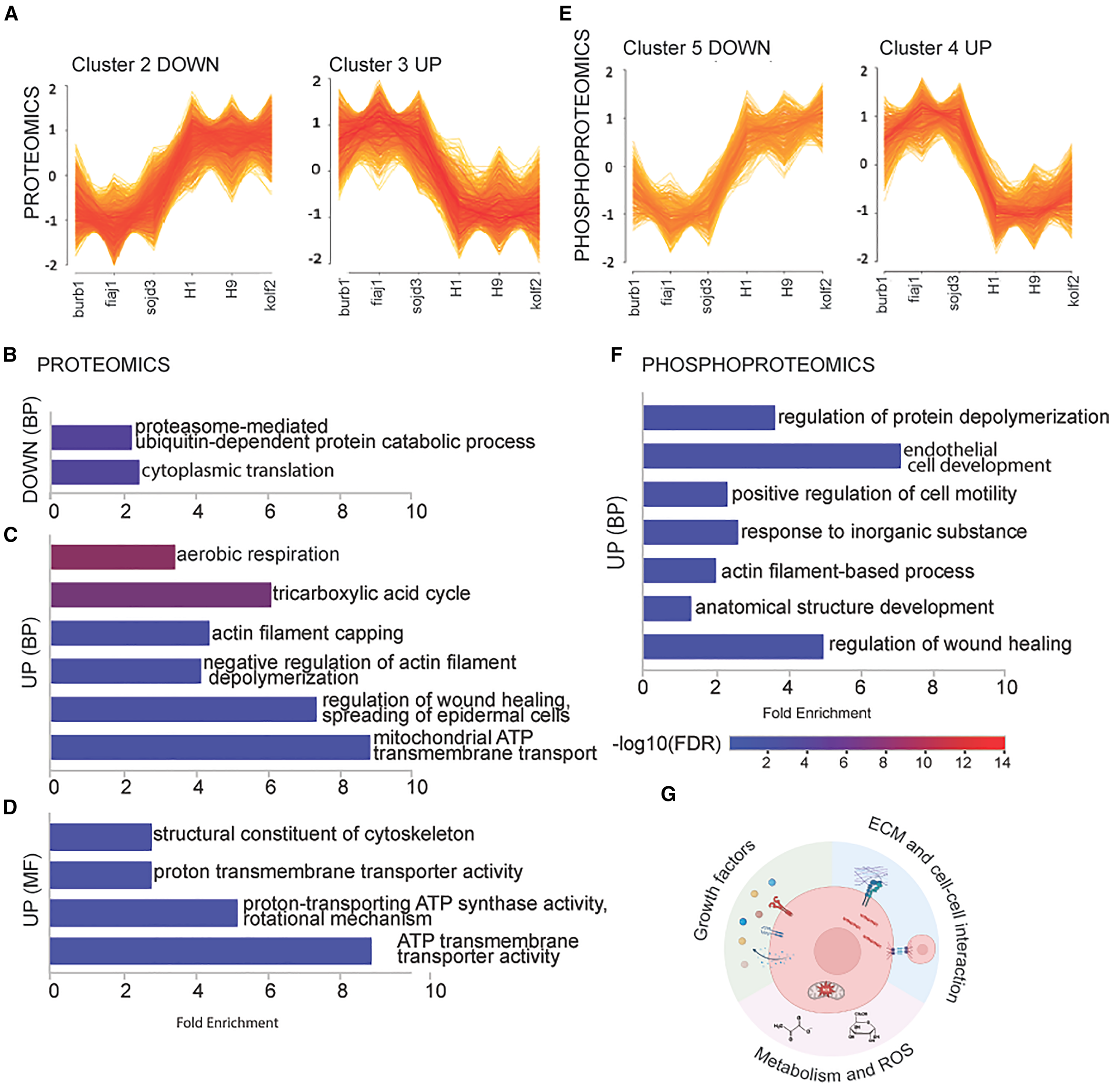

Figure 2. Further proteomic and phosphoproteomics analysis

(A and B) Clusters that distinguish non-competent and competent cells in VSClust analysis of proteome and(B) phosphoproteome.
(C) Selected GO BP terms overrepresented in cluster 2 – proteins lower in non-competent cells.
(D) Selected GO BP terms overrepresented in cluster 3 – proteins higher in non-competent cells.
(E) Selected GO MF terms overrepresented in cluster 3 – proteins higher in non-competent cells.
(F) Selected GO BP terms overrepresented in cluster 4 – phosphoproteins higher in non-competent cells.
(G) Cartoon summary of areas of cell physiology different between competent and non-competent cells. Created in BioRender. Sutcliffe, M. (2026) https://BioRender.com/v4mukb1.

significant results for BP or MF in cluster 5. However, cluster 4 showed one enriched MF (cell adhesion molecule binding) and several enriched BP terms (Figure 2F; Table S8), primarily pointing to the organization of the actin cytoskeleton.

Taken together, these findings suggest that non-competent cell lines upregulate the actin cytoskeleton and oxidative respiration (Figure 2G), providing insights into the molecular underpinnings of cerebral organoid competence.

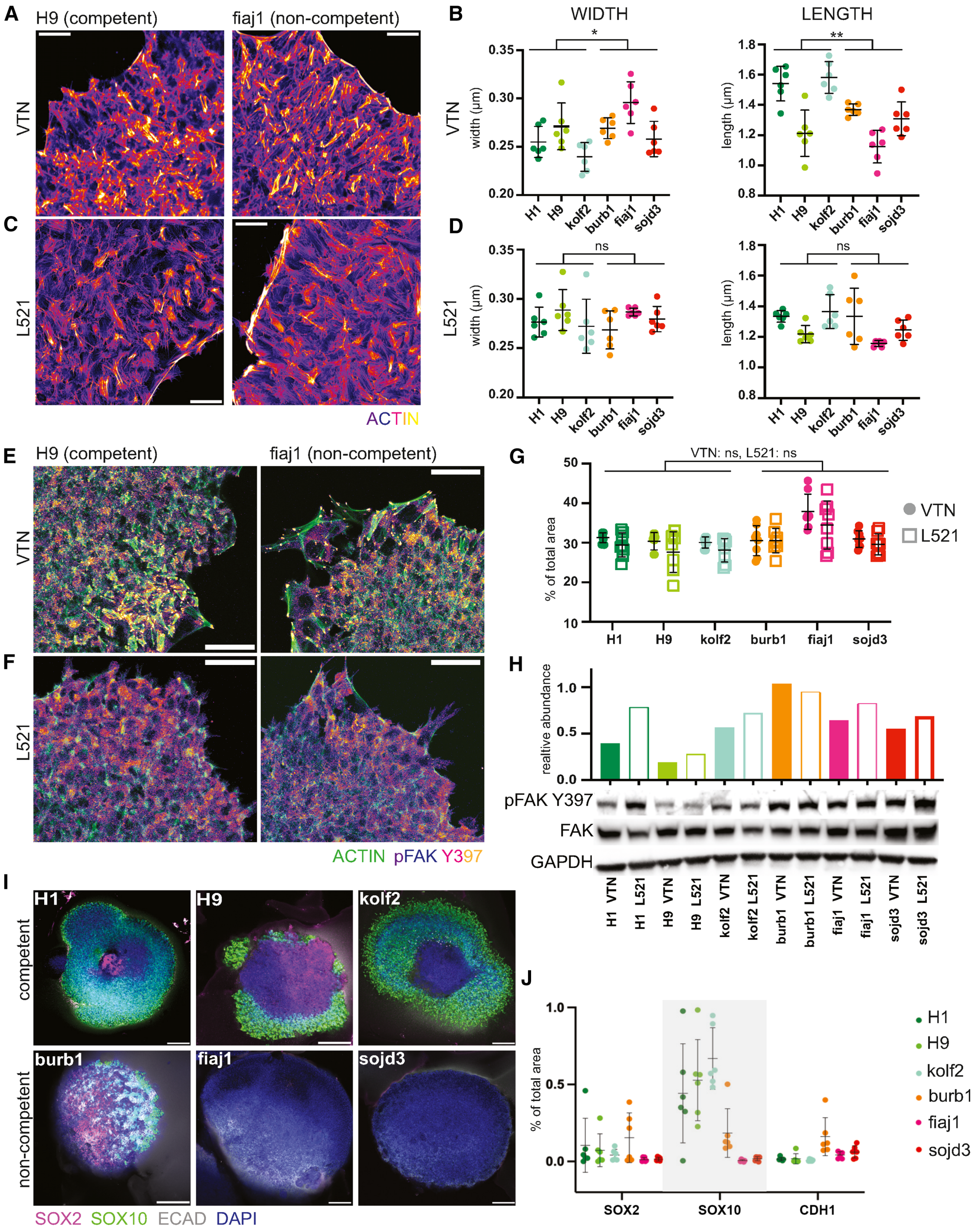

Figure 3. Cytoskeleton and FAs in competency

(A) Phalloidin staining of F-actin in fiaj1 and H9 cells cultured on VTN; scale bars, 100 µm.

(B) Quantification of actin structure morphology on VTN, line shows mean and error bars SD, $n = 6$ areas from 2 batches, Mann-Whitney test, $*p < 0.05$, $**p < 0.01$.

(C) Phalloidin staining of F-actin in fiaj1 and H9 cells cultured on L521; scale bars, 100 µm.

(D) Quantification of actin structure morphology on L521, line shows mean and error bars SD, $n = 6$ areas from 2 batches, Mann-Whitney test, ns $p > 0.05$.

(E) Active focal adhesion kinase and actin staining of fiaj1 and H9 cultured on VTN; scale bars, 50 µm.

(legend continued on next page)

Altered actin cytoskeleton and focal adhesions and the influence of the substrate

We then proceeded to validate the proteomic targets first by staining the actin cytoskeleton in competent and non-competent cell lines to compare their structures (Figure 3A). Quantification of actin structures showed that they tended to be wider (0.2714 vs. 0.2480 μm, $p = 0.0411$) but shorter (1.268 vs. 1.482 μm, $p = 0.0022$) in non-competent lines (Figure 3B).

We then explored whether the morphology of actin in non-competent lines can be modified by the adhesion substrate. Laminin alpha-5 is abundant in the epiblast, and laminins 521 (L521) and 511 are thought to best support an epiblast-like state in PSCs (Albalushi et al., 2018; Laperle et al., 2015). Actin structure morphology changed dramatically in cells cultured on L521 (Figure 3C), whose actin structures no longer differed between non-competent and competent lines (width 0.2803 vs. 0.2777 μm $p > 0.9999$, length 1.219 vs. 1.304, $p = 0.1797$, Figure 3D).

Actin fibers in PSC colonies are typically associated with focal adhesions (FAs), where cells anchor to the extracellular matrix (ECM) and accumulate activated focal adhesion kinase (FAK) Y397 (Närvä et al., 2017). Vitronectin and fibronectin, which engage integrin αvβ5, have been shown to promote the formation of FAs and actin stress fibers in PSCs (Närvä et al., 2017; Stubb et al., 2019). The peripheral adhesions on VTN, so called "cornerstone adhesions," are necessary for maintaining pluripotency, but excess FAs could drive differentiation. On the other hand, L521 mainly engages integrin α6β1, which prevents overactivation of FAK that leads to differentiation (Villa-Diaz et al., 2016).

We stained for phosphorylated FAK (pFAK)-Y397 to determine any differences in FAs between competent and non-competent cells on VTN and L521. FAs in non-competent cells on VTN appeared larger and more abundant than in competent cells (Figure 3E) and became very small and hard to distinguish when cultured on L521 (Figure 3F). However, quantification of pFAK staining did not show significant differences in pFAK-Y397 staining intensity (non-competent vs. competent VTN 30.53% vs. 28.11%, $p = 0.1000$, L521 30.93% vs. 30.38%, $p = 0.4000$, Figure 3G). Similarly, a semi-quantitative analysis by western blot on cells cultured on VTN versus L521 also did not show consistent differences between lines (Figures 3H and S5, supplemental images 1–3).

We then cultured cells on L521-coated dishes to produce cerebral organoids. Organoids grown from burb1 cells on L521 showed slight and inconsistent morphological improvement with some organized areas showing SOX2$^+$ neural progenitors and with only a small portion of SOX10$^+$ (neural crest marker) disorganized tissue (Figure S3A). However, no improvement was seen in organoids from fiaj1 or sojd3 cells, which consisted mostly of SOX10$^+$ cells and did not form neural buds.

We speculated that ECM interaction might be dysregulated in cell lines unresponsive to the switch to L521, potentially hindering neural differentiation. We adapted the PSCs to culture without any ECM in suspension (Li et al., 2018). Cells were dissociated and grown with ROCK inhibitor until they formed small clumps that secreted their own ECM. Our proteomics data confirmed endogenous expression of laminin subunits LAMA1, LAMA5, LAMB1, LAMB2, and LAMC5 in standard conditions, in line with previous reports (Miyazaki et al., 2008; Rodin et al., 2010).

Suspension conditions slightly improved burb1, which showed some areas positive for SOX2 but also large areas positive for SOX10 (Figures 3I and 3J). We did not see improvement in morphology of other non-competent organoids (Figures 3I and 3J). Surprisingly, suspension culture in E8 medium led to deterioration of all the competent lines; H1, H9, and kolf2 showed increased SOX10$^+$ areas and decreased SOX2$^+$ areas. This suggests that cell-ECM interaction is necessary even in naturally competent cells, and although cells can secrete their own ECM, the concentration might not be sufficient to support stemness.

Since we determined that adherent culture on ECM is necessary, but L521 did not reliably improve organoid quality, we speculated that the aberrant ECM interaction in unresponsive cell lines might be the cause. We observed that even on L521, sojd3 and fiaj1 lines tended to spread at the colony periphery (Figure S3B). Peripheral cell spreading makes cells more responsive to endogenous growth factors that prevent neural differentiation (Rosowski et al., 2015; Xue et al., 2018). We hypothesized that restricting cell spreading could make colonies more uniform in their response to differentiation, as previously demonstrated in 2D models of gastrulation (Deglincerti et al., 2016; Warmflash et al., 2014).

To test this, we prepared geometrically constrained circular L521-coated surfaces of approximately 1 mm in diameter for cell attachment (Figure S3C). Once confluent, the micropatterned colonies were then harvested for the generation of cerebral organoids. Immunostaining revealed that

(F) Active focal adhesion kinase and actin staining of fiaj1 and H9 cultured on L521; scale bars, 50 μm.
(G) Quantification of pFAK Y397 staining in lines on VTN and L521, $n = 6$ areas from two batches, Mann-Whitney test, ns $p > 0.05$.
(H) Western blot of active FAK in sample pairs from lines cultured either on VTN or L521.
(I) Day 10 organoids grown from cell lines cultured in suspension culture; scale bars, 200 μm.
(J) Quantification or marker expression in day 10 organoids made from suspension cultures, lines represent means, error bars SD, $n = 6$ organoids from 2 independent batches, Kruskal-Wallis test.

although overall morphology was not improved, a small region with correct morphology and marker expression (SOX2 and TBR2) appeared (Figure S3D) in organoids from otherwise non-competent cells.

In addition to ECM, another potential influence on PSC spreading is media formulation. FGF2 and transforming growth factor β (TGF-β)1 in E8 medium are crucial for maintaining stem cell pluripotency (Vallier et al., 2005), but FGF2 also influences colony morphology (Dvorak et al., 2005). High FGF2 concentration in the media promotes colony compaction and reduces peripheral spreading. E8 medium already has high FGF2 (100 ng/mL [Chen et al., 2011]); however, due to its thermal instability, FGF2 levels decrease by approximately half within the first 4 h of culture (Lotz et al., 2013). To address this problem, we combined L521 coating with a sustained-release FGF2 system (FGF2 DISCs) (Bertucci et al., 2023). We switched the non-competent cell lines to L521/FGF2 DISCs in E8 for at least 2 weeks. We then generated organoids and analyzed them on day 10.

Burb1 produced organoids with extensive areas of correct morphology and a small proportion of $SOX10^+$ cells (Figure S3E). Sojd3 also generated organoids with more $SOX2^+$ cells and localized areas of well-structured buds, though these organoids predominantly contained contaminating $SOX10^+$ tissue. On the other hand, fiaj1 did not exhibit any improvement and displayed the usual rounded morphology with mostly $SOX10^+$ cells. In summary, incorporating sustained-release FGF2 with L521 coating led to somewhat improved morphology in burb1 and sojd3 organoids but did not benefit fiaj1.

Taken together, these data point to the important role of cell substrate and colony morphology in influencing PSC differentiation capacity. We identified improved results with L521 and micropatterned colonies for certain cell lines, while VTN or lack of exogenous ECM altogether negatively impacted differentiation capacity.

Elevated oxidative metabolism in non-competent lines

Another significant finding from our proteomic screen was the upregulation of oxidative metabolism in non-competent cells (Figures 2D and 2E). Non-competent lines upregulated multiple components of complexes I and V of the mitochondrial electron transport chain in Figures 4A and 4B. Human PSCs primarily rely on glycolysis for energy production, converting glucose to pyruvate and then to lactate (Varum et al., 2011). In PSCs, the activity of mitochondrial complex I is low, and oxidative phosphorylation (OxPhos) is suppressed, to limit reactive oxygen species (ROS) generation and support genetic stability (Shetty et al., 2018). Although some pyruvate is directed to the TCA cycle (Zhang et al., 2011), the low expression of aconitase 2 and isocitrate dehydrogenase 2/3 results in citrate being exported to the cytosol (Tohyama et al., 2016). There, it can be converted back to acetyl-CoA by ATP-citrate lyase (ACLY). Acetyl-CoA then serves as a substrate for lipid synthesis and histone acetylation essential for pluripotency maintenance (Moussaieff et al., 2015; Wang et al., 2017).

As such, high OxPhos in the non-competent cell lines is atypical of PSCs and suggests altered mitochondrial physiology (Shetty et al., 2018). To confirm this, we assessed mitochondria morphology in the non-competent and competent lines, both cultured on L521 (Figure 4C). Morphological analysis of the mitochondrial shape revealed that mitochondria in non-competent lines were more rounded (0.7999 vs. 0.7823, $p = 0.148$) and their outline less irregular (0.6115 vs. 0.5452, $p = 0.0003$), which could be indicative of mitochondrial stress (Figure 4D) (Hemel et al., 2025).

Our proteomic data also showed higher expression of pyruvate dehydrogenase E1 subunit beta in non-competent lines suggesting that more pyruvate is directed toward the TCA cycle. We therefore assessed the conversion of glucose to lactate, a key indicator of glycolytic activity. All cell lines exhibited robust conversion of glucose to lactate, with a slightly higher lactate-to-glucose ratio in the competent H9 line (Figure 4E), suggesting a greater reliance on glycolysis than the other cell lines.

Oxygen availability also affects cellular metabolism, and culturing cells under atmospheric oxygen concentrations under a thick layer of medium can lead to local hypoxia due to limited diffusion (Tan et al., 2024). We therefore tested cells cultured in either half-volume media, or half-volume media under hypoxic conditions (5% O_2), and measured glucose and lactate levels after 24 h. Conversion rates were similar in all the conditions tested, suggesting that oxygen concentration or diffusion is not a limiting factor driving metabolic shifts between OxPhos and glycolysis (Figure 4E).

Interestingly, in all PSC lines tested, the ratio of lactate produced to glucose consumed exceeded the expected value of 2, suggesting that PSCs utilize additional substrates from the culture media to produce lactate. Notably, the amount of glucose consumed from the medium was only a small fraction of the total glucose concentration available, implying that with daily medium changes, cells are exposed to unnecessarily high glucose levels (Figure S5A). Excess glucose in culture medium leads to increased respiration rates and results in higher peroxide generation (Crespo et al., 2010). Additionally, E8 medium is lipid-free but contains glutamine, pyruvate, and high levels of insulin and glucose, all of which have been demonstrated to shift the metabolic balance away from glycolysis to the TCA cycle and OxPhos (Chen et al., 2011; Cornacchia et al., 2019; Ren et al., 2020; Song et al., 2019).

To address the supraphysiological glucose concentration in culture media (17 mM), we modified the culture medium

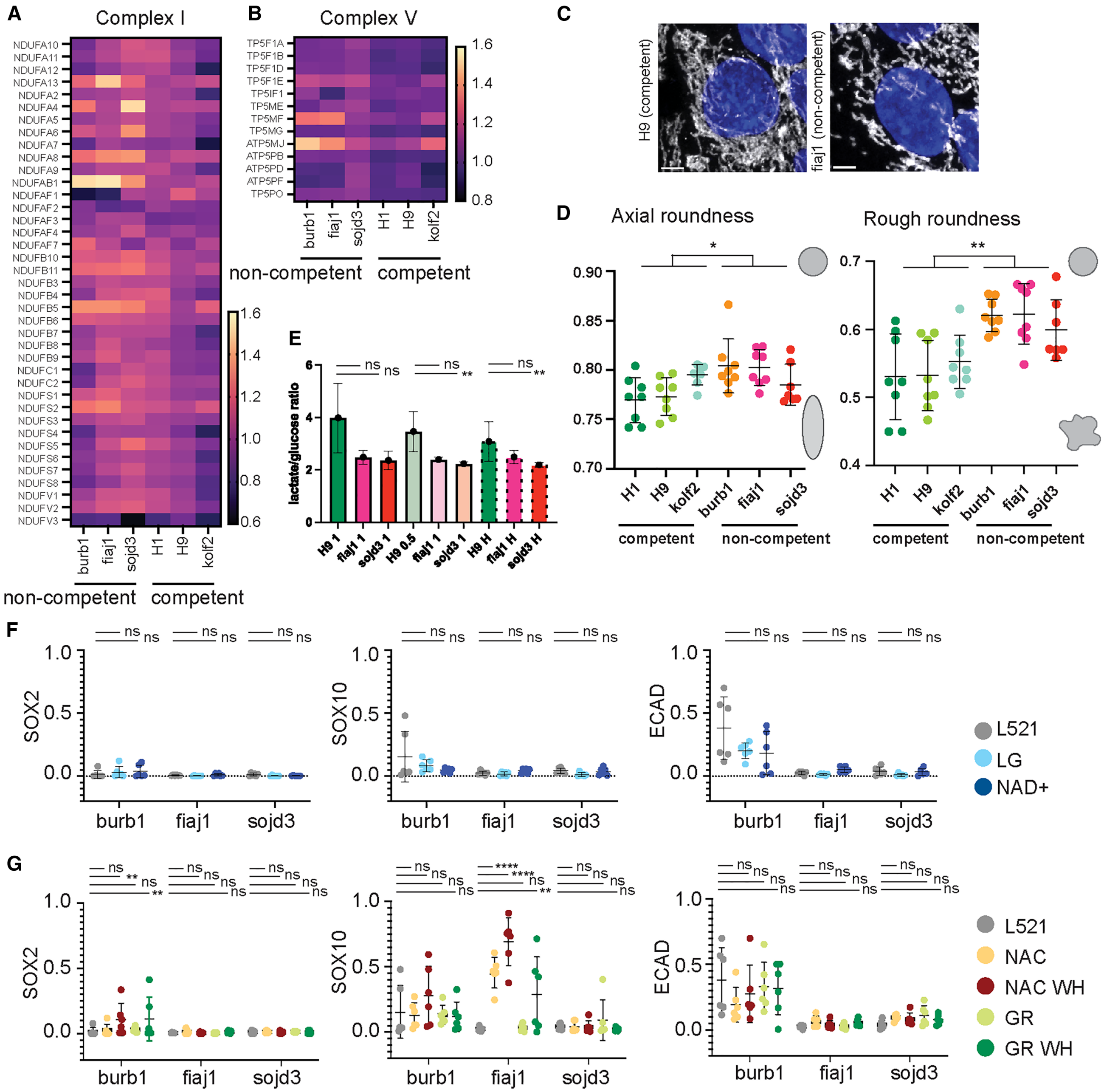

Figure 4. Metabolism in competency

(A and B) Heatmaps of protein abundances of mitochondrial complex I and(B) complex V.

(C) Representative images of mitochondrial morphology in competent and non-competent lines.

(D) Quantification of mitochondrial morphology on L521, line shows mean and error bars SD, n = 6 areas from 2 batches, Mann-Whitney test, $*p < 0.05$, $**p < 0.01$.

(E) Ratio of conversion of glucose to lactate in one competent (H9) and two non-competent lines (fiaj1 and sojd3) in 600 μL of medium (full media labeled 1), 300 μL of medium (half media labeled 0.5), or in 300 μL under hypoxia (5% oxygen, labeled H), error bars show SD, n = 4 batches, Kruskal-Wallis test, ns $p > 0.05$, $**p < 0.01$.

(F) Quantification or marker expression in day 10 organoids made from cultures in E8 on L521, in low-glucose medium or E8 with NAD+, lines represent means, error bars SD, n = 6 organoids from 2 batches, Kruskal-Wallis test, ns $p > 0.05$.

(G) Quantification or marker expression in day 10 organoids made from cultures with combinations of reduced glutathione (GR) and N-acetyl cysteine (NAC) and WH-4-023 (SRC inhibitor), lines represent means, error bars SD, n = 6 organoids from 2 independent batches, Kruskal-Wallis test, ns $p > 0.05$.

with glucose concentration adjusted to 5 mM. We then cultured non-competent lines in this medium for at least 3 passages, generated cerebral organoids, and analyzed them at day 10. Organoids from non-competent lines cultured in low-glucose medium did not show morphological improvement when compared to controls (Figures 4F and S5B).

While OxPhos is superior to glycolysis in terms of ATP generation, glycolysis plays a critical role in regenerating nicotinamide adenine dinucleotide (NAD+) for various metabolic processes and generating molecular building blocks in rapidly proliferating PSCs (Luengo et al., 2021; Zhang et al., 2012). At the same time, directing pyruvate away from lactate generation and into the TCA cycle suppresses cell proliferation by reducing the NAD+/NADH ratio. NAD+ supplementation in human ESCs decreases cellular dependency on glycolysis to maintain a favorable NAD+/NADH ratio, improving pluripotency marker expression (Lees et al., 2020). Since the non-competent cell lines displayed reduced glycolysis, increased TCA activity, and differences in NAD metabolism at the proteomic level (Figure 1D), we hypothesized that they might be depleted in NAD+. To correct the NAD+/NADH ratio, we treated cells with 50 μM NAD+ for at least 3 passages and generated organoids; however, we did not observe improvement in cerebral organoid morphology at day 10 in any of the tested lines (Figures 4F and S5B).

Finally, we explored mitigation of the putative oxidative stress caused by upregulated OxPhos. Supplementation of antioxidants can be as effective as lowering glucose in reducing ROS generation and downstream signaling (Crespo et al., 2010). To test if addition of antioxidants improved organoid competency, we added 2.5 mM of N-acetyl cysteine (NAC) and/or 1 mM reduced glutathione to regular glucose E8 medium. Additionally, we tested combination of antioxidants with targeting FA signaling. We suppressed SRC, a downstream effector of FAK, which itself cannot be inhibited due its role in pluripotency maintenance (Vitillo et al., 2016). WH-4-023, an SRC inhibitor (SRCi), was used during embryoid body (EB) formation, and antioxidants, either alone or with SRCi, were used in PSC culture and for 3 days after EB generation. Antioxidants combined with SRCi showed minimal improvement in marker expression only in burb1 cells, whereas fiaj1 and sojd3 did not improve morphologically (Figures 4G and S5C). Fiaj1 organoids showed increased expression of the SOX10 marker with NAC, whereas burb1 showed expression of CDH1 in all conditions tested.

DISCUSSION

Optimal cell culture conditions of PSCs are crucial for maintenance of their two key features: self-renewal and trilineage differentiation potential (Smith, 2017). Although human ESCs have been cultured for over 25 years now, and iPSCs for almost 20 years, challenges remain to model *in vivo* development. While the self-renewal aspect of PSCs seems to be well understood, differentiation potential appears more problematic (Andrews and Gokhale, 2024; Bock et al., 2011). This limitation is particularly evident in organoid research, where even minor inconsistencies in the starting cell population can be amplified over time, leading to reduced yields or complete differentiation failure. Unguided brain organoids seem to be particularly sensitive to the state of PSCs, whereas their correct complex morphology is crucial to faithfully reflect natural brain development and function (Chiaradia and Lancaster, 2020).

In this study, we delineate proteomic differences associated with organoid competence and demonstrate how optimized PSC culture conditions can enhance this competence. Culture conditions represent the second most influential factor shaping the cellular proteome, after genetic background, and have previously been shown to affect cholesterol biosynthesis, transcription, translation, and vesicular transport (Mirauta et al., 2020). Here, we address two areas of cell physiology highlighted by our proteomic screen, namely dysregulated cell adhesion and an aberrant metabolic shift toward OxPhos in non-competent cell lines. We show that an L521 substrate, steady supply of FGF2, and reducing the impact of increased OxPhos in PSCs were able to increase the proportion of $SOX2^{+}$ neural tissues to some extent and decrease the proportion of other tissues in unguided cerebral organoids.

Three recent studies have demonstrated that the cellular state immediately prior to pluripotency exit is critical for successful cerebral organoid formation (Ideno et al., 2022; Pagliaro et al., 2023; Watanabe et al., 2022). These works collectively highlight the roles of phosphatidylinositol 3-kinase (PI3K) and ERK signaling downstream of FGF2, as well as SMAD activation by TGF-β ligands, with their crosstalk and relative balance determining whether cells maintain pluripotency or commit to differentiation (Singh et al., 2012). Although TGF-β signaling did not emerge from our proteomic screen and was therefore not investigated in PSCs directly, we observed that TGF-β inhibition at the embryoid body stage improved tissue quality (Table 1). With respect to FGF signaling, we found that a sustained supply of FGF2 from DISCs was beneficial, whereas inhibition of FGFR1 or its downstream effectors had limited or negative effects; PI3K inhibition provided only modest benefit, while MEK1/2 inhibition was detrimental (Table 1). The effect of FGF2 supply may be attributed to the stabilization of pathway activity and improved colony morphology (Dvorak et al., 2005; Lotz et al., 2013). Although not tested here, commercially available engineered thermostable variants of FGF2 could yield similar benefits (Dvorak et al., 2018; Onuma et al., 2015).

Table 1. Modifications of cell culture and organoid protocol

Treatment	Rationale and references	Effect	Data shown in this paper
Culture on L521	L521 engages integrin a6 and prevents overactivation of FAK that leads to loss of pluripotency (Villa-Diaz et al., 2016)	improved organoid generation in burb1	yes, Figure S3A
Inhibition of SRC at basal culture (CGP-77675)	inhibition of FAK effector since FAK is needed for pluripotency maintenance (Vitillo et al., 2016) and plays a role in pro-survival signaling through IGF1R (Godoy-Parejo et al., 2019)	deterioration, differentiation of PSC with prolonged exposure	no
Inhibition of SRC at organoid generation (WH-4-023)	suppression of SRC improves differentiation toward all germ layers (Chetty et al., 2015); inhibition caused epithelial differentiation (Lian et al., 2013)	no improvement alone, improves outcome when used alongside antioxidants	yes, Figure 4G
FGF2 DISCs - sustained FGF2 release, 3 feeds a week	FGF2 is crucial for pluripotency (Vallier et al., 2005); sustained release FGF2 from DISCs improves expression of stem cell markers, increases stem cell numbers, and decreases spontaneous differentiation (Lotz et al., 2013)	in cells cultured on L521 improved organoid differentiation in burb1, improved marker expression in sojd3	yes, Figures 3I and 3J
Culture with HRG	FGF2 promotes phosphorylation of ERBB3, an HRG co-receptor (Ding et al., 2011); self-renewal of PSCs requires ERBB2/ERBB3 activation that can be achieved with HRG (Wang et al., 2007)	some improvement in fiaj1, better if combined with antioxidants and/or SRC inhibitor WH-4-023	no
Geometric constriction – culture on L521 micropatterns	culture of PSCs on micropatterns standardizes their response to gastrulation inducing cues *in vitro* (Warmflash et al., 2014); constrained cells in the center of micropatterns are insensitive to WNT signals that induce gastrulation-like process at the colony periphery (Martyn et al., 2019)	some local improvement in fiaj1	yes, Figure S3D
Organoid culture with dual SMAD inhibition	Noggin/SB431542 facilitates neural induction in human PSCs (Chambers et al., 2009); short pulse of Noggin/SB431542 used for 1–3 days in the first 3 days of organoid protocol	only longer treatment shows improvement, leading to change of the character of the differentiation protocol from unguided to guided	no
MEK inhibition in organoids	PD0325901 improves neural ectoderm differentiation from mouse epiblast stem cells (Yu et al., 2018); FGF signaling prevents neural induction through ERK activation in human PSCs (Greber et al., 2011)	some improvement in bad batch of kolf2, no improvement in fiaj1	no

(*Continued on next page*)

Table 1. *Continued*

Treatment	Rationale and references	Effect	Data shown in this paper
Reducing glucose in culture medium	high levels of glucose in standard culture media for PSCs contribute to oxidative stress and bias cells toward mesoderm and cardiomyocyte differentiation (Crespo et al., 2010)	no effect	yes, Figure 4F
Eliminating pyruvate from medium	exogenous pyruvate enhances mesodermal differentiation of PSCs (Song et al., 2019)	no effect	yes, Figure 4F
Long-term culture in E8 medium	culture in E8 leads to improved directed neural differentiation (Cornacchia et al., 2019); E8 medium was used as the default condition in this study	no improvement and in H9 deterioration	yes
Addition of a-ketoglutarate to EBs	alpha-ketoglutarate improves differentiation of primed PSCs though histone modification and DNA methylation (TeSlaa et al., 2016); culture in E8 improves directed neural differentiation through increasing alpha-ketoglutarate/succinate ratio (Cornacchia et al., 2019)	cell death at EB stage	no
Pre-treatment with FGFR1 inhibitor	FGFR1 inhibition 2 days before organoid generation improves identity and morphology to the same extent as culture on feeders (Ideno et al., 2022)	no improvement, in sojd3 deterioration	no
Antioxidants (before and 3 days after EB formation)	high level of oxidative metabolism and ROS bias toward mesoderm differentiation that can be reversed by using antioxidants (Crespo et al., 2010; Song et al., 2019), proteomics results suggest increased mitochondrial oxidative metabolism in bad differentiators	some improvement in burb1, better when combined with SRC inhibitor WH-4-023	yes, Figure 4G
Inhibition of PI3K at EB generation	PI3K drives PSC survival but also increases oxidative metabolism (Ren et al., 2020); phenotypic overlap at the transcriptomic and proteomic level between non-competent and cells with mutant PIK3CA H1047R allele (Madsen et al., 2021), suggesting potential overactivation of the PI3K pathway	addition of PI3K inhibitor at EB generation improved fiaj1	no

We also demonstrate the crucial role of both the presence and the type of ECM in neural competency. Lack of external ECM led to competency loss in previously competent lines, whereas culture on L521, a fully defined, survival and pluripotency-promoting matrix (Albalushi et al., 2018; Rodin et al., 2010), corrected aberrant cytoskeletal morphology and FA organization and improved organoid competency in certain cell lines. In contrast, VTN impaired competency in at least one otherwise competent line. We did not test Matrigel due to its undefined formulation, more promiscuous integrin binding pattern, and potential batch-to-batch variability (Hughes et al., 2010; Meng

et al., 2010). However, its laminin-rich nature may explain why it was reported as a successful substrate for PSCs for cerebral organoid generation (Giandomenico et al., 2021). Although ECM type affected FAs, direct manipulation of FA signaling through inhibition of the downstream effector SRC provided only marginal benefit in one line when applied at pluripotency exit and was generally detrimental to PSC survival (Table 1). Interestingly, several proteins associated with FAs and actin cytoskeleton were previously shown to be upregulated in iPSCs vs. ESCs, pointing to the sensitivity of adhesions to *in vitro* culture conditions (Phanstiel et al., 2011). Nevertheless, global proteomic differences between ESCs and iPSCs remain minimal (Munoz et al., 2011).

We further found that PSC metabolism influences organoid competency, with cell lines exhibiting elevated TCA cycle activity and OxPhos performing worse in organoid differentiation. Consistent with our observations, previous studies have reported that a glycolytic shift enhances the differentiation potential of H9 ESCs (Yamamoto et al., 2022). E8 medium, used here for its simple and fully defined composition, tends to promote more TCA activity and oxidative metabolism in PSCs (Cornacchia et al., 2019). It is plausible that non-competent cells aberrantly upregulate OxPhos in E8 or struggle to manage excess ROS, which could impact their differentiation potential (Crespo et al., 2010; Ji et al., 2010). Although others observed improved directed neural differentiation with E8 medium (Cornacchia et al., 2019), this discrepancy may reflect differences between unguided and directed differentiation, akin to contrasting findings with TGF-β signaling (Bertucci et al., 2023; Watanabe et al., 2022).

A recent comparative proteomic study of iPSCs and ESCs pointed toward metabolic differences between the two PSC types (Brenes et al., 2024). Comparison with our findings revealed that some similarities exist between the features that distinguish ESCs from iPSCs and competent from non-competent cells, but also some important differences. For example, the iPSCs interrogated by Brenes and colleagues (Brenes et al., 2024) upregulated components of all complexes of the electron transport chain, multiple nutrient transporters, and enzymes in key metabolic pathways. However, the non-competent cell lines used here specifically showed higher abundance of complex 1 and 5 proteins. Several of their metabolic hits also showed downregulation in non-competent lines (CPT1A, SLC25A20, ACO2 IDH3A-G, SDHA-B, OGDH, GLUD, GLS, OAT, and GOT2), but some displayed inverse or another pattern (MECR, DLD, and GPT2). This suggests that the metabolic changes might not be iPSC specific but that iPSC lines might have less robust metabolic control, and in suboptimal conditions, some slip into an elevated metabolic state not compatible with competency.

In conclusion, we demonstrate that optimization of culture conditions by seeding on L521 substrate, steady supply of FGF2, and lowering OxPhos or addition of antioxidants can improve cerebral organoid differentiation. Although combinations of these changes produced some degree of improvement in each of the cell lines tested, a universal solution is still lacking, and culture adjustments may need to be tailored and tested for individual lines.

METHODS

Cell and organoid culture

Cells were cultured in E8 medium (Thermo Fisher Scientific) on TC-treated 6-well plates (Corning) coated with rhVTN (Thermo Fisher Scientific) at 20 μg/well or rhL521 at 0.5 mg per well and split twice a week with EDTA.

Cerebral organoids with mainly telencephalic identity cells were generated as described previously using STEMdiff Cerebral Organoid Kit (STEMCELL Technologies, 08570) (Figure 1A) (Lancaster et al., 2017).

Imaging

PFA-fixed frozen samples were prepared and stained as previously described (Lancaster et al., 2013). Images were acquired on Zeiss LSM 710 or Zeiss LSM 780 systems with each channel as separate track at ×100, ×200, or ×630 magnification. Raw images were processed and analyzed using Fiji version 2.16.0/1.54p and Huygens 24.10.0p0 and p5 (Scientific Volume Imaging, the Netherlands, http://svi.nl). Brightness and/or contrast were adjusted where needed for clarity.

Proteomics

Samples were alkylated, digested with trypsin, and labeled with TMT 16plex Isobaric label Reagents (Thermo Scientific). Phosphopeptides were enriched by incubation with TiO_2 beads (Titansphere 10 μm, GL Sciences) and deglycosylated with N-glycosidase F (Biolabs) and sialidase A (Prozyme). Non-modified peptides and phosphopeptides were fractionated by high-pH chromatography prior to reverse-phase nanoLC-MS/MS analysis.

The raw data were processed using Proteome Discoverer (v.2.5, Thermo Fisher Scientific, PD2.5). Quantification across the 2 sets of TMTpro 16-plex was normalized based on a common reference channel containing a mix of all samples. Principal-component analysis and heatmaps were prepared in PD2.5 and Perseus v.1.5.4.1. Differentially abundant peptides were identified by PolyStest applying the Limma test with false discovery rate <0.05 (Schwämmle et al., 2020). Further cluster analysis was performed using variance-sensitive fuzzy clustering (Schwämmle and Jensen, 2018). Hits were evaluated using

Panther (https://pantherdb.org) against the background of all detected peptides. For further details, please refer to supplemental methods.

Immunoblotting

Immunoblotting was performed as described previously (Benito-Kwiecinski et al., 2021). The blots were developed with ECL Prime reagent (GE Healthcare, RPN2232) and imaged using a Chemidoc MP system (Bio-Rad).

Extracellular glucose and lactate measurements

Cells were grown on 24-well plates and fed with 0.6 mL (high) or 0.3 mL (low) of fresh medium. After 24 h, media were collected and analyzed at the Core Biochemical Assay Laboratory (Addenbrooke's Hospital, Cambridge). Fresh medium was used as a baseline. Medium glucose was measured by modified hexokinase-glucose-6-phosphate dehydrogenase method (assay DF30, Siemens Healthcare Diagnostics). Medium lactate was measured by modified Marbach and Weil method (assay DF16, Siemens Healthcare Diagnostics).

RESOURCE AVAILABILITY

Lead contact

Requests for further information and resources should be directed to and will be fulfilled by the lead contact, Madeline A. Lancaster (madeline.lancaster@mrc-lmb.cam.ac.uk).

Materials availability

No new materials were generated in this study.

Data and code availability

The accession number for the mass spectrometry proteomics data is ProteomeXchange Consortium PRIDE (Perez-Riverol et al., 2025): PXD061650.

ACKNOWLEDGMENTS

We thank the MRC Laboratory of Molecular Biology Light Microscopy Facility for technical support. We thank the Core Biochemical Assay Laboratory at Addenbrooke's Hospital, Cambridge, for sample analysis. We thank the members of the Lancaster and Larsen Labs for helpful comments. This work was supported by theMedical Research Council (MC_UP_1201/9) and Medical Research CouncilMDU Mouse Biochemistry Laboratory (MC_UU_00014/5).

AUTHOR CONTRIBUTIONS

M.A.S., M.R.L., and M.A.L. conceived the study, M.A.S. and P.J. performed experiments and analyzed results, C.A.J.M. performed experiments, J.T. analyzed results, D.J.F. provided expertise on glucose metabolism, and M.A.S. and M.A.L. wrote the manuscript.

DECLARATION OF INTERESTS

M.A.L. is an inventor on patents covering cerebral organoids and is a co-founder and advisory board member of a:head bio.

SUPPLEMENTAL INFORMATION

Supplemental information can be found online at https://doi.org/10.1016/j.stemcr.2025.102724.

Received: November 19, 2024
Revised: November 3, 2025
Accepted: November 3, 2025
Published: December 4, 2025

REFERENCES

Albalushi, H., Kurek, M., Karlsson, L., Landreh, L., Kjartansdóttir, K.R., Söder, O., Hovatta, O., and Stukenborg, J.-B. (2018). Laminin 521 Stabilizes the Pluripotency Expression Pattern of Human Embryonic Stem Cells Initially Derived on Feeder Cells. Stem Cells Int. *2018*, 7127042. https://doi.org/10.1155/2018/7127042.

Andrews, P.W., Barbaric, I., Benvenisty, N., Draper, J.S., Ludwig, T., Merkle, F.T., Sato, Y., Spits, C., Stacey, G.N., Wang, H., and Pera, M.F. (2022). The consequences of recurrent genetic and epigenetic variants in human pluripotent stem cells. Cell Stem Cell *29*, 1624–1636. https://doi.org/10.1016/j.stem.2022.11.006.

Andrews, P.W., and Gokhale, P.J. (2024). A short history of pluripotent stem cells markers. Stem Cell Rep. *19*, 1–10. https://doi.org/10.1016/j.stemcr.2023.11.012.

Benito-Kwiecinski, S., Giandomenico, S.L., Sutcliffe, M., Riis, E.S., Freire-Pritchett, P., Kelava, I., Wunderlich, S., Martin, U., Wray, G.A., McDole, K., and Lancaster, M.A. (2021). An early cell shape transition drives evolutionary expansion of the human forebrain. Cell *184*, 2084–2102.e19. https://doi.org/10.1016/j.cell.2021.02.050.

Bertucci, T., Bowles, K.R., Lotz, S., Qi, L., Stevens, K., Goderie, S.K., Borden, S., Oja, L.M., Lane, K., Lotz, R., et al. (2023). *Improved Protocol for Reproducible Human Cortical Organoids Reveals Early Alterations in Metabolism with* MAPT *Mutations*. Preprint at bioRxiv. https://doi.org/10.1101/2023.07.11.548571.

Bock, C., Kiskinis, E., Verstappen, G., Gu, H., Boulting, G., Smith, Z.D., Ziller, M., Croft, G.F., Amoroso, M.W., Oakley, D.H., et al. (2011). Reference Maps of Human ES and iPS Cell Variation Enable High-Throughput Characterization of Pluripotent Cell Lines. Cell *144*, 439–452. https://doi.org/10.1016/j.cell.2010.12.032.

Brenes, A.J., Griesser, E., Sinclair, L.V., Davidson, L., Prescott, A.R., Singh, F., Hogg, E.K.J., Espejo-Serrano, C., Jiang, H., Yoshikawa, H., et al. (2024). Proteomic and functional comparison between human induced and embryonic stem cells. eLife. https://doi.org/10.7554/elife.92025.1.

Brenes, A.J., Yoshikawa, H., Bensaddek, D., Mirauta, B., Seaton, D., Hukelmann, J.L., Jiang, H., Stegle, O., and Lamond, A.I. (2021). Erosion of human X chromosome inactivation causes major remodeling of the iPSC proteome. Cell Rep. *35*, 109032. https://doi.org/10.1016/j.celrep.2021.109032.

Camp, J.G., Badsha, F., Florio, M., Kanton, S., Gerber, T., Wilsch-Bräuninger, M., Lewitus, E., Sykes, A., Hevers, W., Lancaster, M., et al. (2015). Human cerebral organoids recapitulate gene expression programs of fetal neocortex development. Proc. Natl. Acad. Sci. USA *112*, 15672–15677. https://doi.org/10.1073/pnas.1520760112.

Chambers, S.M., Fasano, C.A., Papapetrou, E.P., Tomishima, M., Sadelain, M., and Studer, L. (2009). Highly efficient neural conversion of human ES and iPS cells by dual inhibition of SMAD signaling. Nat. Biotechnol. *27*, 275–280. https://doi.org/10.1038/nbt.1529.

Chen, G., Gulbranson, D.R., Hou, Z., Bolin, J.M., Ruotti, V., Probasco, M.D., Smuga-Otto, K., Howden, S.E., Diol, N.R., Propson, N.E., et al. (2011). Chemically defined conditions for human iPSC derivation and culture. Nat. Methods *8*, 424–429. https://doi.org/10.1038/nmeth.1593.

Chetty, S., Engquist, E.N., Mehanna, E., Lui, K.O., Tsankov, A.M., and Melton, D.A. (2015). A Src inhibitor regulates the cell cycle of human pluripotent stem cells and improves directed differentiation. J. Cell Biol. *210*, 1257–1268. https://doi.org/10.1083/jcb.201502035.

Chiaradia, I., and Lancaster, M.A. (2020). Brain organoids for the study of human neurobiology at the interface of in vitro and in vivo. Nat. Neurosci. *23*, 1496–1508. https://doi.org/10.1038/s41593-020-00730-3.

Cornacchia, D., Zhang, C., Zimmer, B., Chung, S.Y., Fan, Y., Soliman, M.A., Tchieu, J., Chambers, S.M., Shah, H., Paull, D., et al. (2019). Lipid Deprivation Induces a Stable, Naive-to-Primed Intermediate State of Pluripotency in Human PSCs. Cell Stem Cell *25*, 120–136.e10. https://doi.org/10.1016/j.stem.2019.05.001.

Crespo, F.L., Sobrado, V.R., Gomez, L., Cervera, A.M., and McCreath, K.J. (2010). Mitochondrial reactive oxygen species mediate cardiomyocyte formation from embryonic stem cells in high glucose. Stem Cell. *28*, 1132–1142. https://doi.org/10.1002/stem.441.

Deglincerti, A., Etoc, F., Guerra, M.C., Martyn, I., Metzger, J., Ruzo, A., Simunovic, M., Yoney, A., Brivanlou, A.H., Siggia, E., and Warmflash, A. (2016). Self-organization of human embryonic stem cells on micropatterns. Nat. Protoc. *11*, 2223–2232. https://doi.org/10.1038/nprot.2016.131.

Ding, V.M.Y., Boersema, P.J., Foong, L.Y., Preisinger, C., Koh, G., Natarajan, S., Lee, D.-Y., Boekhorst, J., Snel, B., Lemeer, S., et al. (2011). Tyrosine phosphorylation profiling in FGF-2 stimulated human embryonic stem cells. PLoS One *6*, e17538. https://doi.org/10.1371/journal.pone.0017538.

Dvorak, P., Bednar, D., Vanacek, P., Balek, L., Eiselleova, L., Stepankova, V., Sebestova, E., Kunova Bosakova, M., Konecna, Z., Mazurenko, S., et al. (2018). Computer-assisted engineering of hyperstable fibroblast growth factor 2. Biotechnol. Bioeng. *115*, 850–862. https://doi.org/10.1002/bit.26531.

Dvorak, P., Dvorakova, D., Koskova, S., Vodinska, M., Najvirtova, M., Krekac, D., and Hampl, A. (2005). Expression and Potential Role of Fibroblast Growth Factor 2 and Its Receptors in Human Embryonic Stem Cells. STEM CELLS *23*, 1200–1211. https://doi.org/10.1634/stemcells.2004-0303.

Giandomenico, S.L., Sutcliffe, M., and Lancaster, M.A. (2021). Generation and long-term culture of advanced cerebral organoids for studying later stages of neural development. Nat. Protoc. *16*, 579–602. https://doi.org/10.1038/s41596-020-00433-w.

Glass, M.R., Waxman, E.A., Yamashita, S., Lafferty, M., Beltran, A.A., Farah, T., Patel, N.K., Singla, R., Matoba, N., Ahmed, S., et al. (2024). Cross-site reproducibility of human cortical organoids reveals consistent cell type composition and architecture. Stem Cell Rep. *19*, 1351–1367. https://doi.org/10.1016/j.stemcr.2024.07.008.

Godoy-Parejo, C., Deng, C., Liu, W., and Chen, G. (2019). Insulin Stimulates PI3K/AKT and Cell Adhesion to Promote the Survival of Individualized Human Embryonic Stem Cells. Stem Cell. *37*, 1030–1041. https://doi.org/10.1002/stem.3026.

Greber, B., Coulon, P., Zhang, M., Moritz, S., Frank, S., Müller-Molina, A.J., Araúzo-Bravo, M.J., Han, D.W., Pape, H.-C., and Schöler, H.R. (2011). FGF signalling inhibits neural induction in human embryonic stem cells. EMBO J. *30*, 4874–4884. https://doi.org/10.1038/emboj.2011.407.

Hemel, I.M.G.M., Knoops, K., López-Iglesias, C., and Gerards, M. (2025). The Curse of the Red Pearl: A Fibroblast-Specific Pearl-Necklace Mitochondrial Phenotype Caused by Phototoxicity. Biomolecules *15*, 304. https://doi.org/10.3390/biom15020304.

Hughes, C.S., Postovit, L.M., and Lajoie, G.A. (2010). Matrigel: A complex protein mixture required for optimal growth of cell culture. Proteomics *10*, 1886–1890. https://doi.org/10.1002/pmic.200900758.

Ideno, H., Imaizumi, K., Shimada, H., Sanosaka, T., Nemoto, A., Kohyama, J., and Okano, H. (2022). Human PSCs determine the competency of cerebral organoid differentiation via FGF signaling and epigenetic mechanisms. iScience *25*, 105140. https://doi.org/10.1016/j.isci.2022.105140.

Jerber, J., Seaton, D.D., Cuomo, A.S.E., Kumasaka, N., Haldane, J., Steer, J., Patel, M., Pearce, D., Andersson, M., Bonder, M.J., et al. (2021). Population-scale single-cell RNA-seq profiling across dopaminergic neuron differentiation. Nat. Genet. *53*, 304–312. https://doi.org/10.1038/s41588-021-00801-6.

Ji, A.-R., Ku, S.-Y., Cho, M.S., Kim, Y.Y., Kim, Y.J., Oh, S.K., Kim, S.H., Moon, S.Y., and Choi, Y.M. (2010). Reactive oxygen species enhance differentiation of human embryonic stem cells into mesendodermal lineage. Exp. Mol. Med. *42*, 175–186. https://doi.org/10.3858/emm.2010.42.3.018.

Kilpinen, H., Goncalves, A., Leha, A., Afzal, V., Alasoo, K., Ashford, S., Bala, S., Bensaddek, D., Casale, F.P., Culley, O.J., et al. (2017). Common genetic variation drives molecular heterogeneity in human iPSCs. Nature *546*, 370–375. https://doi.org/10.1038/nature22403.

Lancaster, M.A., Corsini, N.S., Wolfinger, S., Gustafson, E.H., Phillips, A.W., Burkard, T.R., Otani, T., Livesey, F.J., and Knoblich, J.A. (2017). Guided self-organization and cortical plate formation in human brain organoids. Nat. Biotechnol. *35*, 659–666. https://doi.org/10.1038/nbt.3906.

Lancaster, M.A., and Knoblich, J.A. (2014). Generation of cerebral organoids from human pluripotent stem cells. Nat. Protoc. *9*, 2329–2340. https://doi.org/10.1038/nprot.2014.158.

Lancaster, M.A., Renner, M., Martin, C.-A., Wenzel, D., Bicknell, L.S., Hurles, M.E., Homfray, T., Penninger, J.M., Jackson, A.P., and Knoblich, J.A. (2013). Cerebral organoids model human brain development and microcephaly. Nature *501*, 373–379. https://doi.org/10.1038/nature12517.

Laperle, A., Hsiao, C., Lampe, M., Mortier, J., Saha, K., Palecek, S.P., and Masters, K.S. (2015). α-5 Laminin Synthesized by Human Pluripotent Stem Cells Promotes Self-Renewal. Stem Cell Rep. *5*, 195–206. https://doi.org/10.1016/j.stemcr.2015.06.009.

Lees, J.G., Gardner, D.K., and Harvey, A.J. (2020). Nicotinamide adenine dinucleotide induces a bivalent metabolism and maintains pluripotency in human embryonic stem cells. Stem Cell. *38*, 624–638. https://doi.org/10.1002/stem.3152.

Lian, X., Selekman, J., Bao, X., Hsiao, C., Zhu, K., and Palecek, S.P. (2013). A small molecule inhibitor of SRC family kinases promotes simple epithelial differentiation of human pluripotent stem cells. PLoS One *8*, e60016. https://doi.org/10.1371/journal.pone.0060016.

Lippmann, E.S., Estevez-Silva, M.C., and Ashton, R.S. (2014). Defined Human Pluripotent Stem Cell Culture Enables Highly Efficient Neuroepithelium Derivation Without Small Molecule Inhibitors. Stem Cell. *32*, 1032–1042. https://doi.org/10.1002/stem.1622.

Lotz, S., Goderie, S., Tokas, N., Hirsch, S.E., Ahmad, F., Corneo, B., Le, S., Banerjee, A., Kane, R.S., Stern, J.H., et al. (2013). Sustained levels of FGF2 maintain undifferentiated stem cell cultures with biweekly feeding. PLoS One *8*, e56289. https://doi.org/10.1371/journal.pone.0056289.

Luengo, A., Li, Z., Gui, D.Y., Sullivan, L.B., Zagorulya, M., Do, B.T., Ferreira, R., Naamati, A., Ali, A., Lewis, C.A., et al. (2021). Increased demand for NAD+ relative to ATP drives aerobic glycolysis. Mol. Cell *81*, 691–707.e6. https://doi.org/10.1016/j.molcel.2020.12.012.

Madsen, R.R., Longden, J., Knox, R.G., Robin, X., Völlmy, F., Macleod, K.G., Moniz, L.S., Carragher, N.O., Linding, R., Vanhaesebroeck, B., and Semple, R.K. (2021). NODAL/TGFβ signalling mediates the self-sustained stemness induced by PIK3CAH1047R homozygosity in pluripotent stem cells. Dis. Model. Mech. *14*, dmm048298. https://doi.org/10.1242/dmm.048298.

Martyn, I., Brivanlou, A.H., and Siggia, E.D. (2019). A wave of WNT signaling balanced by secreted inhibitors controls primitive streak formation in micropattern colonies of human embryonic stem cells. Development (Cambridge, England) *146*, dev172791. https://doi.org/10.1242/dev.172791.

Meng, Y., Eshghi, S., Li, Y.J., Schmidt, R., Schaffer, D.V., and Healy, K.E. (2010). Characterization of integrin engagement during defined human embryonic stem cell culture. FASEB J. *24*, 1056–1065. https://doi.org/10.1096/fj.08-126821.

Merkle, F.T., Ghosh, S., Genovese, G., Handsaker, R.E., Kashin, S., Meyer, D., Karczewski, K.J., O'Dushlaine, C., Pato, C., Pato, M., et al. (2022). Whole-genome analysis of human embryonic stem cells enables rational line selection based on genetic variation. Cell Stem Cell *29*, 472–486.e7. https://doi.org/10.1016/j.stem.2022.01.011.

Mirauta, B.A., Seaton, D.D., Bensaddek, D., Brenes, A., Bonder, M.J., Kilpinen, H., HipSci Consortium, Stegle, O., Lamond, A.I., Danecek, P., et al. (2020). Population-scale proteome variation in human induced pluripotent stem cells. eLife *9*, e57390. https://doi.org/10.7554/elife.57390.

Miyazaki, T., Futaki, S., Hasegawa, K., Kawasaki, M., Sanzen, N., Hayashi, M., Kawase, E., Sekiguchi, K., Nakatsuji, N., and Suemori, H. (2008). Recombinant human laminin isoforms can support the undifferentiated growth of human embryonic stem cells. Biochem. Biophys. Res. Commun. *375*, 27–32. https://doi.org/10.1016/j.bbrc.2008.07.111.

Moussaieff, A., Rouleau, M., Kitsberg, D., Cohen, M., Levy, G., Barasch, D., Nemirovski, A., Shen-Orr, S., Laevsky, I., Amit, M., et al. (2015). Glycolysis-Mediated Changes in Acetyl-CoA and Histone Acetylation Control the Early Differentiation of Embryonic Stem Cells. Cell Metab. *21*, 392–402. https://doi.org/10.1016/j.cmet.2015.02.002.

Munoz, J., Low, T.Y., Kok, Y.J., Chin, A., Frese, C.K., Ding, V., Choo, A., and Heck, A.J.R. (2011). The quantitative proteomes of human-induced pluripotent stem cells and embryonic stem cells. Mol. Syst. Biol. *7*, 550. https://doi.org/10.1038/msb.2011.84.

Närvä, E., Stubb, A., Guzmán, C., Blomqvist, M., Balboa, D., Lerche, M., Saari, M., Otonkoski, T., and Ivaska, J. (2017). A Strong Contractile Actin Fence and Large Adhesions Direct Human Pluripotent Colony Morphology and Adhesion. Stem Cell Rep. *9*, 67–76. https://doi.org/10.1016/j.stemcr.2017.05.021.

Onuma, Y., Higuchi, K., Aiki, Y., Shu, Y., Asada, M., Asashima, M., Suzuki, M., Imamura, T., and Ito, Y. (2015). A Stable Chimeric Fibroblast Growth Factor (FGF) Can Successfully Replace Basic FGF in Human Pluripotent Stem Cell Culture. PLoS One *10*, e0118931. https://doi.org/10.1371/journal.pone.0118931.

Pagliaro, A., Finger, R., Zoutendijk, I., Bunschuh, S., Clevers, H., Hendriks, D., and Artegiani, B. (2023). Temporal morphogen gradient-driven neural induction shapes single expanded neuroepithelium brain organoids with enhanced cortical identity. Nat. Commun. *14*, 7361. https://doi.org/10.1038/s41467-023-43141-1.

Pantazis, C.B., Yang, A., Lara, E., McDonough, J.A., Blauwendraat, C., Peng, L., Oguro, H., Kanaujiya, J., Zou, J., Sebesta, D., et al. (2022). A reference human induced pluripotent stem cell line for large-scale collaborative studies. Cell Stem Cell *29*, 1685–1702.e22. https://doi.org/10.1016/j.stem.2022.11.004.

Perez-Riverol, Y., Bandla, C., Kundu, D.J., Kamatchinathan, S., Bai, J., Hewapathirana, S., John, N.S., Prakash, A., Walzer, M., Wang, S., and Vizcaíno, J.A. (2025). The PRIDE database at 20 years: 2025 update. Nucleic Acids Res. *53*, D543–D553. https://doi.org/10.1093/nar/gkae1011.

Phanstiel, D.H., Brumbaugh, J., Wenger, C.D., Tian, S., Probasco, M.D., Bailey, D.J., Swaney, D.L., Tervo, M.A., Bolin, J.M., Ruotti, V., et al. (2011). Proteomic and phosphoproteomic comparison of human ES and iPS cells. Nat. Methods *8*, 821–827. https://doi.org/10.1038/nmeth.1699.

Pollen, A.A., Bhaduri, A., Andrews, M.G., Nowakowski, T.J., Meyerson, O.S., Mostajo-Radji, M.A., Di Lullo, E., Alvarado, B., Bedolli, M., Dougherty, M.L., et al. (2019). Establishing Cerebral Organoids as Models of Human-Specific Brain Evolution. Cell *176*, 743–756.e17. https://doi.org/10.1016/j.cell.2019.01.017.

Puigdevall, P., Jerber, J., Danecek, P., Castellano, S., and Kilpinen, H. (2023). Somatic mutations alter the differentiation outcomes of iPSC-derived neurons. Cell Genom. *3*, 100280. https://doi.org/10.1016/j.xgen.2023.100280.

Ren, Z., Zhong, H., Song, C., Deng, C., Hsieh, H.-T., Liu, W., and Chen, G. (2020). Insulin Promotes Mitochondrial Respiration and Survival through PI3K/AKT/GSK3 Pathway in Human Embryonic Stem Cells. Stem Cell Rep. *15*, 1362–1376. https://doi.org/10.1016/j.stemcr.2020.10.008.

Renner, M., Lancaster, M.A., Bian, S., Choi, H., Ku, T., Peer, A., Chung, K., and Knoblich, J.A. (2017). Self-organized developmental patterning and differentiation in cerebral organoids. EMBO J. *36*, 1316–1329. https://doi.org/10.15252/embj.201694700.

Rodin, S., Domogatskaya, A., Ström, S., Hansson, E.M., Chien, K.R., Inzunza, J., Hovatta, O., and Tryggvason, K. (2010). Long-term self-renewal of human pluripotent stem cells on human recombinant laminin-511. Nat. Biotechnol. *28*, 611–615. https://doi.org/10.1038/nbt.1620.

Rosowski, K.A., Mertz, A.F., Norcross, S., Dufresne, E.R., and Horsley, V. (2015). Edges of human embryonic stem cell colonies display distinct mechanical properties and differentiation potential. Sci. Rep. *5*, 14218. https://doi.org/10.1038/srep14218.

Sandoval, S.O., Cappuccio, G., Kruth, K., Osenberg, S., Khalil, S.M., Méndez-Albelo, N.M., Padmanabhan, K., Wang, D., Niciu, M.J., Bhattacharyya, A., et al. (2024). Rigor and reproducibility in human brain organoid research: Where we are and where we need to go. Stem Cell Rep. *19*, 796–816. https://doi.org/10.1016/j.stemcr.2024.04.008.

Schwämmle, V., Hagensen, C.E., Rogowska-Wrzesinska, A., and Jensen, O.N. (2020). PolySTest: Robust Statistical Testing of Proteomics Data with Missing Values Improves Detection of Biologically Relevant Features. Mol. Cell. Proteomics *19*, 1396–1408. https://doi.org/10.1074/mcp.RA119.001777.

Schwämmle, V., and Jensen, O.N. (2018). VSClust: Feature-based variance-sensitive clustering of omics data. Bioinformatics *34*, 2965–2972. https://doi.org/10.1093/bioinformatics/bty224.

Shetty, D.K., Kalamkar, K.P., and Inamdar, M.S. (2018). OCIAD1 Controls Electron Transport Chain Complex I Activity to Regulate Energy Metabolism in Human Pluripotent Stem Cells. Stem Cell Rep. *11*, 128–141. https://doi.org/10.1016/j.stemcr.2018.05.015.

Singh, A.M., Reynolds, D., Cliff, T., Ohtsuka, S., Mattheyses, A.L., Sun, Y., Menendez, L., Kulik, M., and Dalton, S. (2012). Signaling Network Crosstalk in Human Pluripotent Cells: A Smad2/3-Regulated Switch that Controls the Balance between Self-Renewal and Differentiation. Cell Stem Cell *10*, 312–326. https://doi.org/10.1016/j.stem.2012.01.014.

Smith, A. (2017). Formative pluripotency: The executive phase in a developmental continuum. Development *144*, 365–373. https://doi.org/10.1242/dev.142679.

Song, C., Xu, F., Ren, Z., Zhang, Y., Meng, Y., Yang, Y., Lingadahalli, S., Cheung, E., Li, G., Liu, W., et al. (2019). Elevated Exogenous Pyruvate Potentiates Mesodermal Differentiation through Metabolic Modulation and AMPK/mTOR Pathway in Human Embryonic Stem Cells. Stem Cell Rep. *13*, 338–351. https://doi.org/10.1016/j.stemcr.2019.06.003.

Stubb, A., Guzmán, C., Närvä, E., Aaron, J., Chew, T.-L., Saari, M., Miihkinen, M., Jacquemet, G., and Ivaska, J. (2019). Superresolution architecture of cornerstone focal adhesions in human pluripotent stem cells. Nat. Commun. *10*, 4756. https://doi.org/10.1038/s41467-019-12611-w.

Takahashi, K., and Yamanaka, S. (2006). Induction of Pluripotent Stem Cells from Mouse Embryonic and Adult Fibroblast Cultures by Defined Factors. Cell *126*, 663–676. https://doi.org/10.1016/j.cell.2006.07.024.

Tan, J., Virtue, S., Norris, D.M., Conway, O.J., Yang, M., Bidault, G., Gribben, C., Lugtu, F., Kamzolas, I., Krycer, J.R., et al. (2024). Limited oxygen in standard cell culture alters metabolism and function of differentiated cells. EMBO J. *43*, 2127–2165. https://doi.org/10.1038/s44318-024-00084-7.

TeSlaa, T., Chaikovsky, A.C., Lipchina, I., Escobar, S.L., Hochedlinger, K., Huang, J., Graeber, T.G., Braas, D., and Teitell, M.A. (2016). α-Ketoglutarate Accelerates the Initial Differentiation of Primed Human Pluripotent Stem Cells. Cell Metab. *24*, 485–493. https://doi.org/10.1016/j.cmet.2016.07.002.

Thomson, J.A., Itskovitz-Eldor, J., Shapiro, S.S., Waknitz, M.A., Swiergiel, J.J., Marshall, V.S., and Jones, J.M. (1998). Embryonic Stem Cell Lines Derived from Human Blastocysts. Science *282*, 1145–1147.

Tohyama, S., Fujita, J., Hishiki, T., Matsuura, T., Hattori, F., Ohno, R., Kanazawa, H., Seki, T., Nakajima, K., Kishino, Y., et al. (2016). Glutamine Oxidation Is Indispensable for Survival of Human Pluripotent Stem Cells. Cell Metab. *23*, 663–674. https://doi.org/10.1016/j.cmet.2016.03.001.

Vallier, L., Alexander, M., and Pedersen, R.A. (2005). Activin/Nodal and FGF pathways cooperate to maintain pluripotency of human embryonic stem cells. J. Cell Sci. *118*, 4495–4509. https://doi.org/10.1242/jcs.02553.

Varum, S., Rodrigues, A.S., Moura, M.B., Momcilovic, O., Easley, C.A., Ramalho-Santos, J., Van Houten, B., and Schatten, G. (2011). Energy Metabolism in Human Pluripotent Stem Cells and Their Differentiated Counterparts. PLoS One *6*, e20914. https://doi.org/10.1371/journal.pone.0020914.

Velasco, S., Kedaigle, A.J., Simmons, S.K., Nash, A., Rocha, M., Quadrato, G., Paulsen, B., Nguyen, L., Adiconis, X., Regev, A., et al. (2019). Individual brain organoids reproducibly form cell diversity of the human cerebral cortex. Nature *570*, 523–527. https://doi.org/10.1038/s41586-019-1289-x.

Villa-Diaz, L.G., Kim, J.K., Laperle, A., Palecek, S.P., and Krebsbach, P.H. (2016). Inhibition of Focal Adhesion Kinase Signaling by Integrin α6β1 Supports Human Pluripotent Stem Cell Self-Renewal. Stem Cell. *34*, 1753–1764. https://doi.org/10.1002/stem.2349.

Vitillo, L., Baxter, M., Iskender, B., Whiting, P., and Kimber, S.J. (2016). Integrin-Associated Focal Adhesion Kinase Protects Human Embryonic Stem Cells from Apoptosis, Detachment, and Differentiation. Stem Cell Rep. *7*, 167–176. https://doi.org/10.1016/j.stemcr.2016.07.006.

Wang, L., Schulz, T.C., Sherrer, E.S., Dauphin, D.S., Shin, S., Nelson, A.M., Ware, C.B., Zhan, M., Song, C.-Z., Chen, X., et al. (2007). Self-renewal of human embryonic stem cells requires insulin-like growth factor-1 receptor and ERBB2 receptor signaling. Blood *110*, 4111–4119. https://doi.org/10.1182/blood-2007-03-082586.

Wang, L., Zhang, T., Wang, L., Cai, Y., Zhong, X., He, X., Hu, L., Tian, S., Wu, M., Hui, L., et al. (2017). Fatty acid synthesis is critical for stem

cell pluripotency via promoting mitochondrial fission. EMBO J. *36*, 1330–1347. https://doi.org/10.15252/embj.201695417.

Warmflash, A., Sorre, B., Etoc, F., Siggia, E.D., and Brivanlou, A.H. (2014). A method to recapitulate early embryonic spatial patterning in human embryonic stem cells. Nat. Methods *11*, 847–854. https://doi.org/10.1038/nmeth.3016.

Watanabe, M., Buth, J.E., Haney, J.R., Vishlaghi, N., Turcios, F., Elahi, L.S., Gu, W., Pearson, C.A., Kurdian, A., Baliaouri, N.V., et al. (2022). TGFβ superfamily signaling regulates the state of human stem cell pluripotency and capacity to create well-structured telencephalic organoids. Stem Cell Rep. *17*, 2220–2238. https://doi.org/10.1016/j.stemcr.2022.08.013.

Wataya, T., Ando, S., Muguruma, K., Ikeda, H., Watanabe, K., Eiraku, M., Kawada, M., Takahashi, J., Hashimoto, N., and Sasai, Y. (2008). Minimization of exogenous signals in ES cell culture induces rostral hypothalamic differentiation. Proc. Natl. Acad. Sci. USA *105*, 11796–11801. https://doi.org/10.1073/pnas.0803078105.

Xue, X., Sun, Y., Resto-Irizarry, A.M., Yuan, Y., Aw Yong, K.M., Zheng, Y., Weng, S., Shao, Y., Chai, Y., Studer, L., and Fu, J. (2018). Mechanics-guided embryonic patterning of neuroectoderm tissue from human pluripotent stem cells. Nat. Mater. *17*, 633–641. https://doi.org/10.1038/s41563-018-0082-9.

Yamamoto, T., Arita, M., Kuroda, H., Suzuki, T., and Kawamata, S. (2022). Improving the differentiation potential of pluripotent stem cells by optimizing culture conditions. Sci. Rep. *12*, 14147. https://doi.org/10.1038/s41598-022-18400-8.

Ying, Q.-L., Stavridis, M., Griffiths, D., Li, M., and Smith, A. (2003). Conversion of embryonic stem cells into neuroectodermal precursors in adherent monoculture. Nat. Biotechnol. *21*, 183–186. https://doi.org/10.1038/nbt780.

Yu, Y., Wang, X., Zhang, X., Zhai, Y., Lu, X., Ma, H., Zhu, K., Zhao, T., Jiao, J., Zhao, Z.-A., and Li, L. (2018). ERK inhibition promotes neuroectodermal precursor commitment by blocking self-renewal and primitive streak formation of the epiblast. Stem Cell Res. Ther. *9*, 2. https://doi.org/10.1186/s13287-017-0750-8.

Zhang, J., Khvorostov, I., Hong, J.S., Oktay, Y., Vergnes, L., Nuebel, E., Wahjudi, P.N., Setoguchi, K., Wang, G., Do, A., et al. (2011). UCP2 regulates energy metabolism and differentiation potential of human pluripotent stem cells: UCP2 regulates hPSC metabolism and differentiation. EMBO J. *30*, 4860–4873. https://doi.org/10.1038/emboj.2011.401.

Zhang, J., Nuebel, E., Daley, G.Q., Koehler, C.M., and Teitell, M.A. (2012). Metabolic Regulation in Pluripotent Stem Cells during Reprogramming and Self-Renewal. Cell Stem Cell *11*, 589–595. https://doi.org/10.1016/j.stem.2012.10.005.

Li, X., Ma, R., Gu, Q., Liang, L., Wang, L., Zhang, Y., Wang, X., Liu, X., Li, Z., Fang, J., et al. (2018). A fully defined static suspension culture system for large-scale human embryonic stem cell production. Cell Death & Disease *9*, 892. https://doi.org/10.1038/s41419-018-0863-8.

Stem Cell Reports, Volume *21*

Supplemental Information

Adjusting PSC culture for neural organoid generation

Magdalena A. Sutcliffe, Pia Jensen, Joycelyn Tan, Charles A.J. Morris, Daniel J. Fazakerley, Martin R. Larsen, and Madeline A. Lancaster

Supplemental Materials

Supplemental Figures

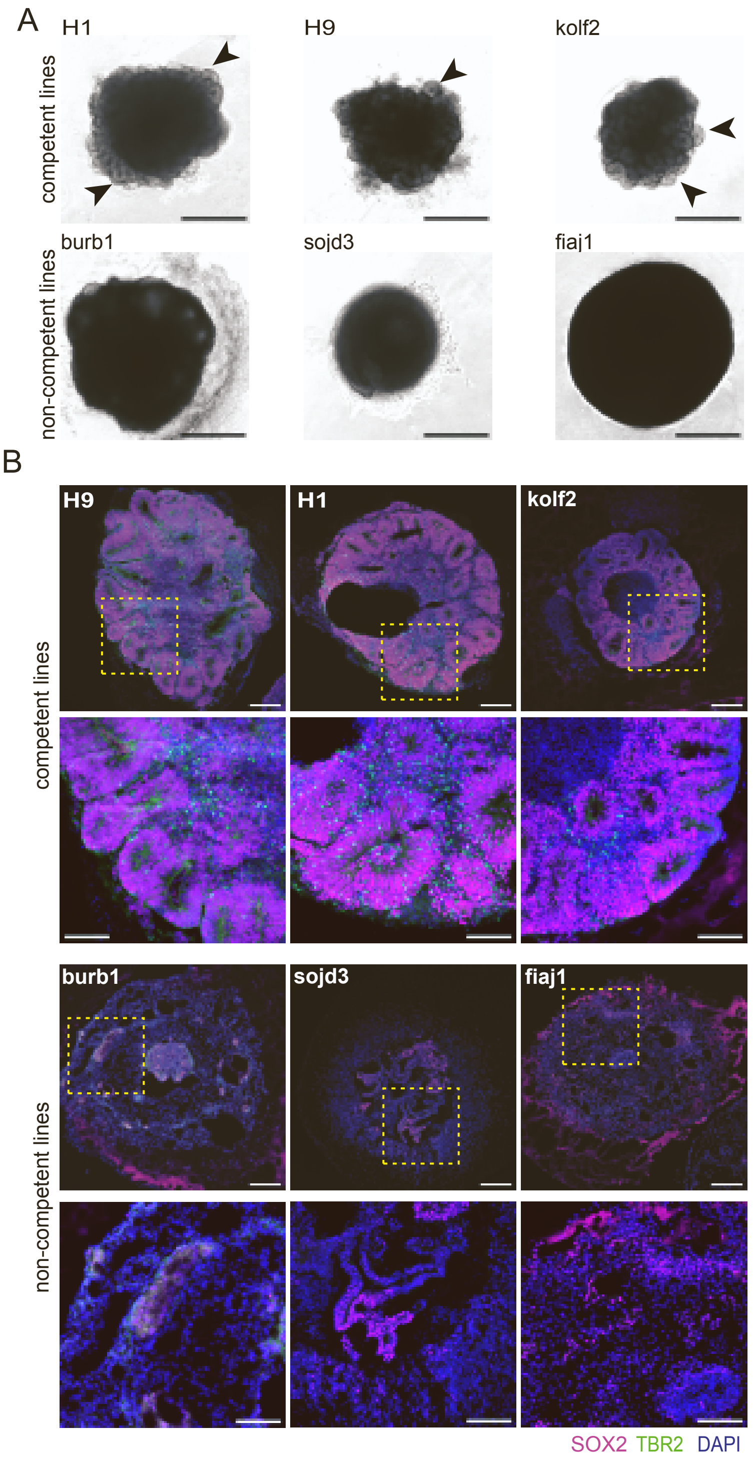

Figure S1. Morphology of organoids from competent and non-competent lines, related to Figure 1

A – representative bright field images of day 10 organoids, arrows indicate neuroepithelial buds, scale bars 500μm, B - representative images of day 20 organoids made from a competent and non-competent lines, scale bar 200 μm (upper panel) or 100 μm (bottom panel) for each line

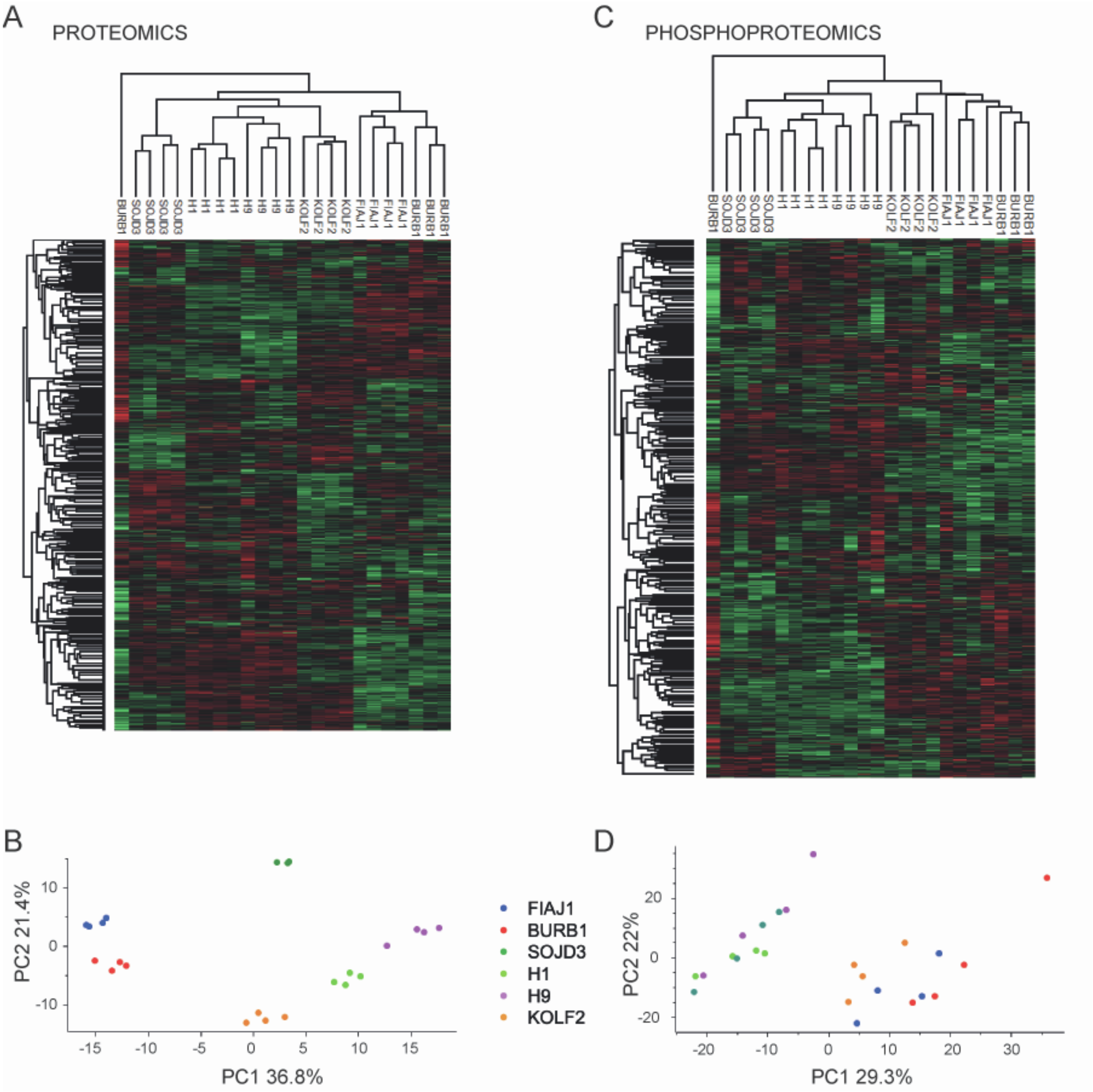

<u>Fig S2 Proteomic sample characterisation</u>

A – principal component analysis based on 5,798 non-modified proteins detected, plot shows PC1 vs PC2, B - heat map of the relative expression levels of the non -modified proteins, C - principal component analysis based on 12,143 phospho-peptides detected, plot shows PC1 vs PC2, D - heat map of the relative expression levels of phosphopeptides

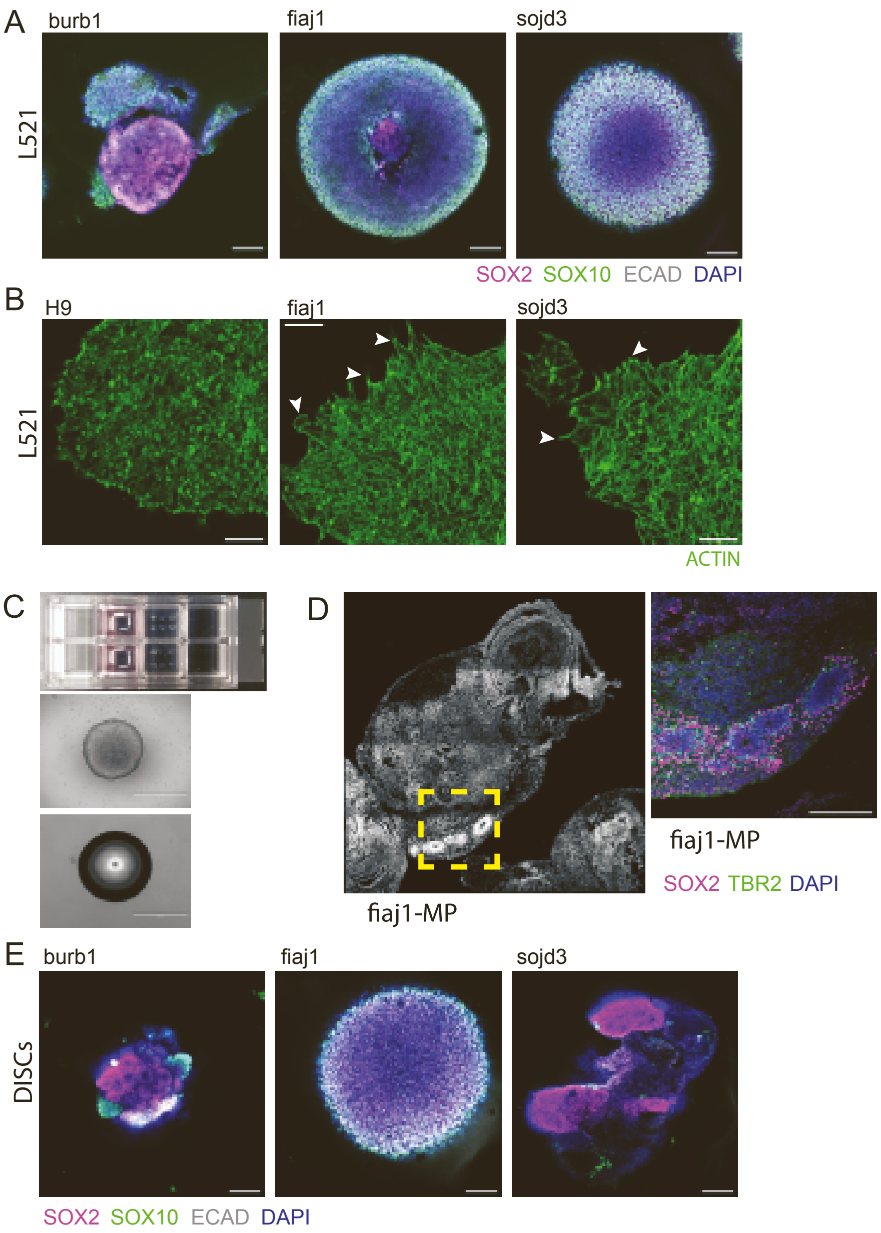

<u>Fig S3 Differences in response to treatments in non-competent lines, related to Figure 3</u>

A - day 10 organoids grown from cells cultured on L521, scale bars 200µm, B – preparation of micropattern colonies – droplets of L521 on a 8-well imaging slide, a droplet of L521 under phase contrast, and an attached micropatterned colony, scale bars 1000µm, C – overview and area of day20 fiaj1 organoids with morphology typical of a competent line, scale bar 200µm, D – day 10 organoids grown from cells cultured on L521 with FGF2 DISCs, scale bars 200µm

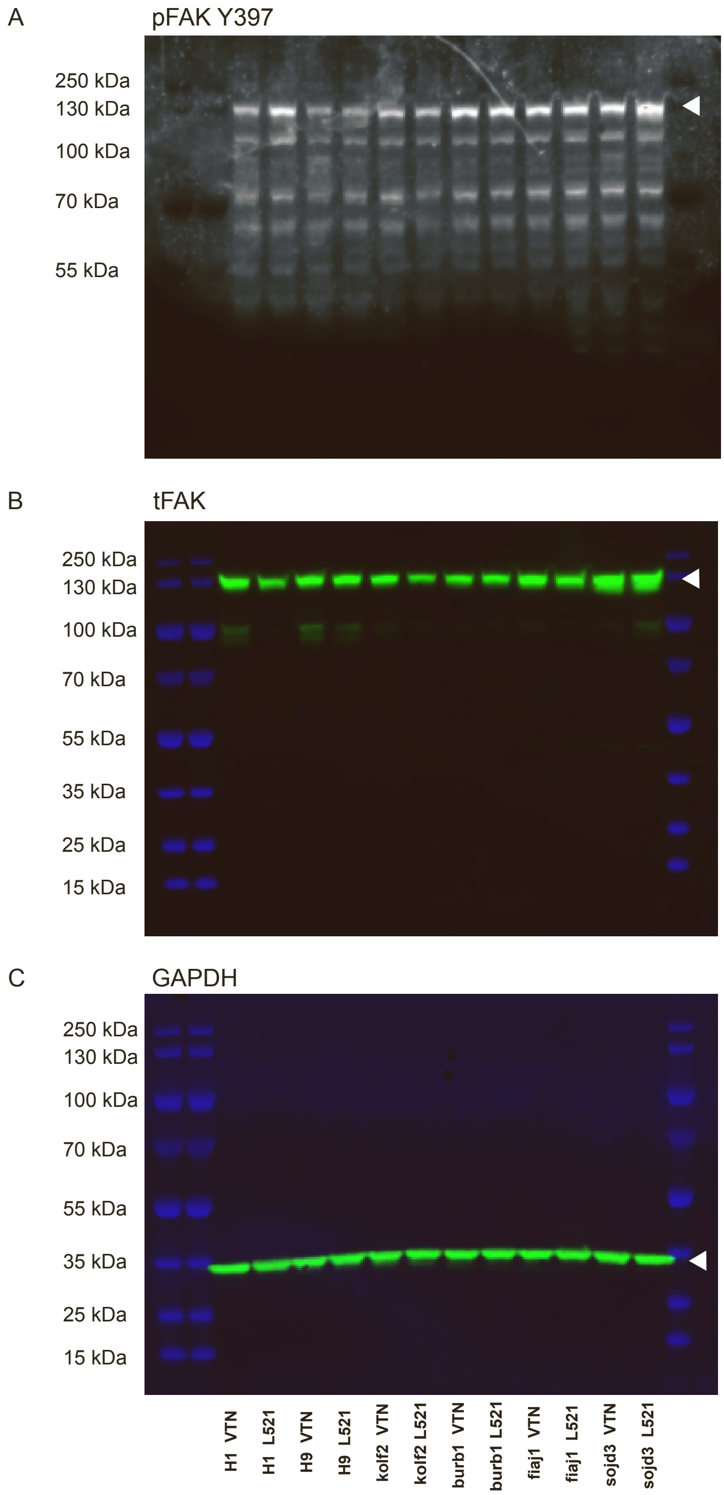

<u>Fig S4 Full size unedited Western blot scans, relating to Figure 3</u>

A – FAK phospho Y397, B – total FAK, C – GAPDH, white arrow heads show the correct band

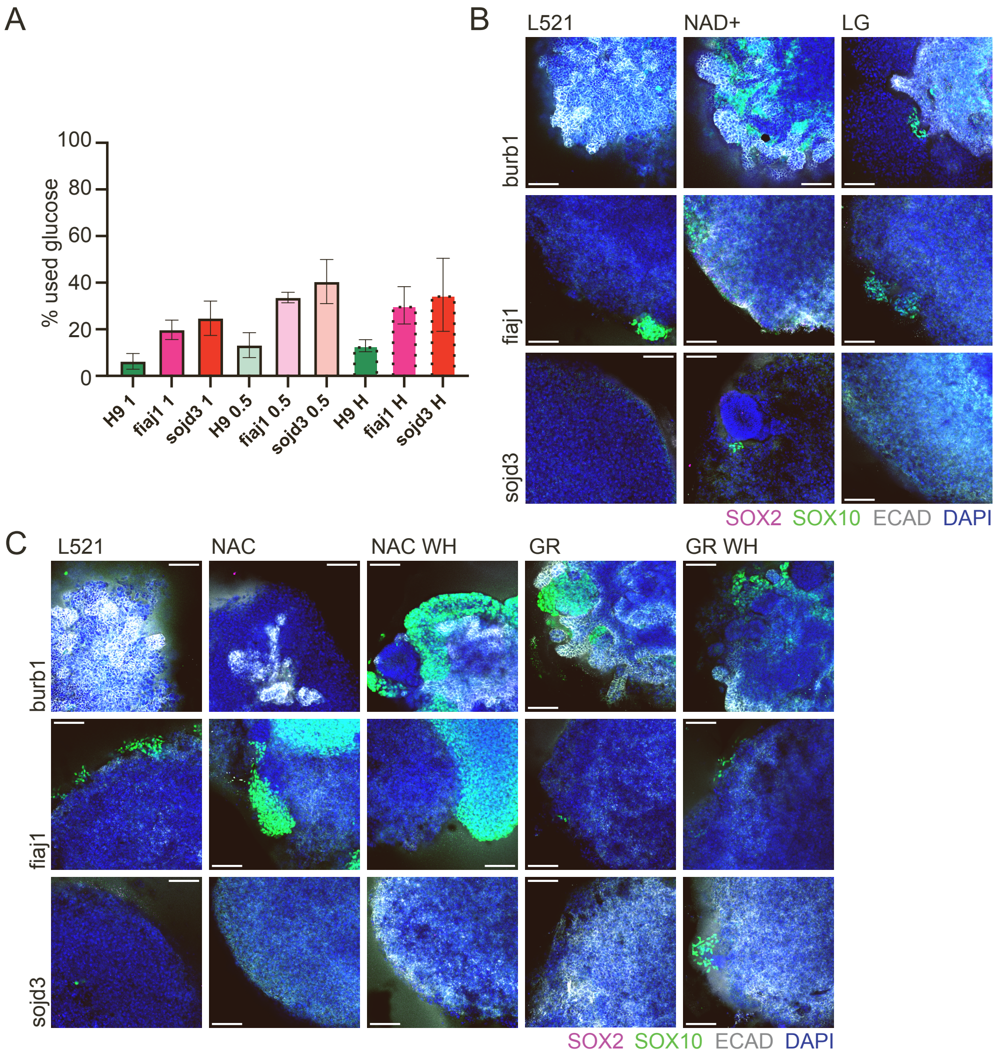

Fig S5 Manipulation of metabolic differences in non-competent lines, relating to Figure 4

A - glucose consumption after 24h, refers to Fig 4E, B – representative images of day 10 organoids made from cultures in E8 on L521, in low glucose medium or E8 supplemented with NAD+, scale bars 100µm, refers to Fig 4F, C - day10 organoids made from cultures with combinations of (GR) reduced glutathione and (NAC) N-acetyl cysteine and WH-4-023 (SRC inhibitor), lines represent means, bars 100µm, refers to Fig 4G

Supplemental Table Legends

All tables in separate .xlsx files

Supplemental Table S1. Quantification of images of day 20 organoids, related to Figure 1

Supplemental Table S2. Abundance of peptides in all samples (proteomics), related to Figure 1.

Supplemental Table S3. Abundance of peptides in all samples (phosphoproteomics), related to Figure 1.

Supplemental Table S4. Differentially abundant peptides, related to Figure 1. List of statistically differentially abundant peptides in proteomics data (Sheet1), in bold hits with fold change >1.5 and full results (Sheet2), condition 1 competent lines (H1, H9, kolf2), condition 2 non-competent lines (burb1, fiaj1, sojd3). List of statistically differentially abundant peptides in phosphoproteomics data (Sheet3), in bold hits with fold change >1.5 and full results (Sheet4), condition 1 competent lines (H1, H9, kolf2), condition 2 non-competent lines (burb1, fiaj1, sojd3).

Supplemental Table S5. Gene Ontology analysis of differentially abundant peptides, related to Figure 1. Data analysed using online tool pantherdb.org against all Homo sapiens genes, Fischer test with FDR correction. Sheet1 – proteomics upregulated in non-competent, Biological Process, Sheet2 - proteomics downregulated in non-competent, Biological Process, Sheet3 – phosphoproteomics upregulated in non-competent, Sheet4 - phosphoproteomics downregulated in non-competent

Supplemental Table S6. Variance Sensitive Fuzzy Clustering (VSClust) of the proteome data, relates to Figure 2.

Supplemental Table S7. Variance Sensitive Fuzzy Clustering (VSClust) of the phosphoproteome data, relates to Figure 2.

Supplemental Table S8. Gene Ontology analysis of differentially abundant peptides identified through Variance Sensitive Fuzzy Clustering (VSClust), relates to Figure 2. Sheet 1 – Cluster 2 unmodified peptides less abundant in non-competent, enriched GO Biological Process terms, Sheet 2 – Cluster 3 unmodified peptides more abundant in non-competent, enriched GO Biological Process terms, Sheet 3 – Cluster 3 unmodified peptides more abundant in non-competent, enriched GO Molecular Function terms, Sheet 4 - Cluster 4 phosphopeptides more abundant in non-competent, enriched GO Biological Process terms, Sheet 5 - Cluster 4 phosphopeptides more abundant in non-competent, enriched GO Molecular Function terms,

Supplemental experimental procedures

Cell lines

The ESC lines used in this study were H9 (WA09, female) and H1 (WA01, male) and were purchased from WiCell. The iPSC lines: burb1 (HPSI0714i-burb_1, hPSCreg WTSIi257-A, male), fiaj1 (HPSI0514i-fiaj_1, hPSCreg WTSIi301-A male), kolf2 (HPSI0114i-kolf_2, hPSCreg WTSIi018-B, male) and sojd3 (HPSI0314i-sojd_3, hPSCreg WTSIi073-A, female) were obtained from hispci.org. The master banks of iPSC lines were verified to be pluripotent in Pluritest and karyotyped by the vendor. The use of human ESCs used for this project was approved by the U.K. Stem Cell Bank Steering Committee and iPSCs and ESCs were approved by an ERC ethics committee and are registered on the Human Pluripotent StemCell Registry (hpscreg.eu).

Cell culture

All lines were grown from working cell banks of less than passage 50 and were cultured for less than 25 subsequent passages. All cell lines were cultured in Essential 8 (E8) medium (Thermo Fisher Scientific, A1517001) on TC-treated 6 well plates (Corning, 3516) coated with rh-VTN (Thermo Fischer Scientific, A14700) at 10 µg/ well. Cultures were split as clumps twice a week using 0.5mM EDTA at the ration of 1:4 to 1:10. When needed, cryopreservation was performed using mFreSR™ (StemCell Technologies, 05855). All cells were cultured without antibiotics or antimycotics to allow visualization of any potential contamination and if observed, cells were immediately discarded. Cells were routinely tested for mycoplasma using MycoAlert™ mycoplasma detection kit (Lonza, LT07). Where specified, culture plates were coated with rhL521 (Thermo Fisher Scientific, A29249) at 5 µg per well in DPBS with calcium and magnesium. For some experiments, cells were cultured in low glucose/low insulin medium, consisting of DMEM no glucose no pyruvate (Thermo Fisher Scientific, A1443001), Sodium bicarbonate 7.5%, L-Ascorbic acid 2-phosphate sesquimagnesium salt hydrate (Merck, A8960-5G), B27-A (Thermo Fisher Scientific, 17504044), N2 (Thermo Fisher Scientific, 17502001), MEMNEAA (Thermo Fisher Scientific, 11140050), Glucose 5mM, and growth factors FGF2 (100ng/mL, Peprotech, 100-18B-50UG) and TGFb (2 µg/mL Gibco, 100-21-10UG). This modified medium did not contain pyruvate, contained antioxidants from B27-A (DL Alpha Tocopherol Acetate, DL Alpha-Tocopherol, reduced glutathione, catalase and superoxide dismutase) and contained lower concentrations of insulin (5.3mg/L vs 19.4mg/mL) and glucose (5mM vs 17.5mM) than E8 medium. Geometrically constrained surfaces for cell attachment on glass slides (Ibidi, 80807) were prepared by applying droplets of L521 mixed 1:1 with DPBS containing calcium and magnesium (Gibco, 14040117). Gentle pipetting of a 0.5 µL portion of this solution produced a droplet of approximately 1 mm in diameter, forming an adhesive field for cells to attach (Fig S3 C). 250,000 single cells were seeded in each well with ROCK inhibitor (Y27632, Santa Cruz, sc-281642), allowed to attach for 2h and then washed to remove unattached cells.

Cerebral organoid generation

Cerebral organoids with telencephalic identity cells were generated using STEMdiff Cerebral Organoid Kit (StemCell Technologies, 08570), Fig 1A) according to manufacturer's protocol. Briefly, cultures at <80% confluence were washed with PBS and dissociated using Accutase (Gibco, A1110501). 9,000 cells were seeded in each well of a round bottom ultra-low adhesion 96 well plate (Corning) in EB medium with 10 µM Y27632 and left for 3 days to form embryoid bodies. Medium was replaced on day 3 with fresh EB medium without Y27632 and then changed to NI medium on day 5. EBs were embedded in Matrigel (Corning, 356235) droplets on day 7 and transferred to Expansion medium. On day 10 droplets were transferred to Maturation Medium. On day 13 Matrigel was removed using mechanical Dissociation and/or 30 minute incubation with Cell Recovery Solution (Corning, 354253) at 4°C, then tissues were transferred to fresh Maturation Medium and cultured with agitation.

Histological and immunohistochemical analysis

Samples were fixed in 4% PFA either overnight at 4 °C or at room temperature for 1h, then washed twice in PBS for 10 min. Samples for cryosectioning were incubated overnight in 30% sucrose in 0.2M PB (21.8 g/L Na2HPO4, 6.4 g/L NaH2PO4 in dH2O), embedded in gelatine (7.5% gelatine, 10% sucrose in 0.2 M PB), and plunge frozen in 2-methylbutane (Sigma-Aldrich, M32631) at below -30 °C. Frozen blocks were sectioned at the thickness of 20 µm and stained. Wholemount samples were stained for 24-48h with primary antibodies followed by 24-48 h with secondary antibodies in a buffer of 4% normal donkey serum and 0.25% Triton-X-100 in PBS.

Antibodies and stains

Primary antibodies used in this study were as follows: SOX2 (Abcam, ab97959, 1:200 for IF), TBR2 (R&D Systems, AF6166 1:200 for IF), HuC/D (Invitrogen, A2127, 1:200 for IF), FAK (Abcam, ab40794, 1:1000 for WB), pFAKY397 (Thermo Fisher Scientific, 44-624G, 1:200 for IF, 1:1000 for WB), GAPDH (Abcam, ab8245, 1:5000 for WB), SOX10 (R&D Systems, AF2864, 1:100-1:200 for IF), ECAD (BD Transduction, 610181, 1:400 for IF), TOM20 (Santa Cruz, sc-17764 1:500 for IF). Secondary antibodies used were: Donkey a-Ms 488 (Life Technologies, A21202), Donkey a-Rb 488 (Life Technologies, A21206), Donkey a-Sh 488 (Life Technologies, A11015), Donkey a-Ms 568 (Life Technologies, A10037), Donkey a-Rb 568 (Life Technologies, A10042), Donkey a-Ms 647 (Life Technologies, A31571), Donkey a-Rb 647 (Life Technologies, A31573), Donkey a-Sh 647 (Life Technologies, A21448). All secondary antibodies were used at 1:500 dilution. Nuclei were counterstained with 0.1 µg/mL DAPI (Merck, 268298). F-actin was detected with ActinGreen 488 ReadyProbes Reagent (Alexa fluor 488 conjugated phalloidin, Invitrogen).

Imaging and image analysis

Images were acquired on Zeiss LSM 710 or Zeiss LSM 780 systems with each channel as separate track. Images were acquired at x100, x200 or x630 magnification. Raw images were processed using FIJI and brightness and/or contrast were adjusted where needed for clarity.
Actin structure quantification: Prior to image analysis all images were deconvolved with Huygens Professional Medium version 24.10.0p0 (Scientific Volume Imaging, The Netherlands, http://svi.nl), using the CMLE algorithm, with following parameters -it 30 -q 0.01 -snr 19.73 -acuity 0.00 -acuityMode on -bg 0.0 -bgMode lowest -bgRadius 0.7 -pad auto -mode fast -reduceMode auto -blMode off -brMode auto -varPsf off -tclReturn. Deconvolved images were processed in FIJI Version 2.16.0/1.54p using top hat filter with radius 4, then threshold default dark to create a binary mask. The “analyse particles” function was applied with a minimum size of 0.10 and the major and minor axis parameters were saved as length and width, respectively.
Quantification of pFAK immunofluorescence: Images were deconvolved as described above and processed in FIJI Version 2.16.0/1.54p. Areas where cells were present in an image were detected based on the DAPI channel using Image>Adjust>Threshold with Default settings, adjusted manually and saved as DAPI ROI. Within DAPI ROI fluorescent signal was isolated using Image>Adjust>Threshold with Default settings and the % of total area was calculated as (signal area)/(DAPI ROI area).
Quantification of organoid morphology: Images were processed in FIJI Version 2.16.0/1.54p. A single image per organoid was quantified. Total area of organoid optical section was detected based on the DAPI channel using Image>Adjust>Threshold with Default settings, adjusted manually and saved as DAPI ROI. Within DAPI ROI fluorescent signal was isolated using Image>Adjust>Threshold with Default settings and the % of total area was calculated as (signal area)/(DAPI ROI area).
Quantification of mitochondria morphology: Prior to image analysis all images were deconvolved with Huygens Professional Medium version 24.10.0p5 (Scientific Volume Imaging, The Netherlands, http://svi.nl), using the CMLE algorithm, with following parameters -it 20 -q 0.01 -snr 19.86 -acuity 0.00 -acuityMode on -bg 0.0 -bgMode lowest -bgRadius 0.7 -pad auto -mode fast -reduceMode auto -blMode off -brMode auto -varPsf off -tclReturn. Mitochondria were segmented using Huygens Object Analyzer function with the following parameters: -gaussMode 0, -threshAbs 13.5, -seed 37.65, -

garbage 100, -segMode watershed, -fragmentation 100, -seedMode sparse and small particles geometry report was generated with axial roundness and rough roundness values used for analysis.

Statistical analysis

Data were collected across two independent batches of organoids or cells. The number of samples, “n” was 6 for all the experiments except for protemics. For cell experiments “n” represents an area analysed and all measurements of individual structures were averaged per area. For organoid experiments “n” represents individual organoids. for Data were analysed in Prism version Version 10.4.1 and 10.4.2. Where comparisons were made between competent and non-competent cells, a Mann-Whitney test was applied and the values analysed were the means in each group. Where comparisons were made between different treatments, and in glucose to lactate conversion experiments, a Kruskal-Wallis test was used. Data are presented as individual datapoints with lines representing mean+/-SD, except from figures 4E and S4A where data are presented as columns representing mean and error bars representing SD.

Protein extraction, digestion and Tandem Mass Tag labeling

At the point of organoid generation, 60% of Accutase dissociated cells were pelleted at 200rcf, the supernatant was removed, and the remaining pellets were frozen at -70°C. Material was collected from 4 independent organoid batches for each cell line. Cell pellets were dissolved in 150 µL lysis buffer consisting of 1% (w/v) sodium deoxycholate (SDC, Sigma), 10 mM Dithiothreitol (DTT, Sigma), and 50 mM triethylammonium bicarbonate (TEAB, Sigma) at pH 8 and sonicated on ice 4x for 10s using a probe sonicator at 40% amplitude. Protein concentrations were measured using a NanoPhotometer® N60 (Implen) against a standard curve of HeLa cell protein extract. 50 µg of each sample was alkylated with 20 mM Iodoacetamide (IAA, Sigma) for 30 minutes in the dark followed by digestion with 5% (w/w) in-house methylated trypsin (Sigma)(Heissel et al., 2018) for 4h at 37°C.
Thereafter the peptide samples were labelled with Tandem Mass Tag (TMT) 16plex Isobaric label Reagents (Thermo Scientific) according to the manufacturer's instructions.
Two sets of a TMTpro 16-plex were used, in each TMT set one channel was used for labelling of a pooled sample to enable comparison of samples between the TMT sets. The labelling reaction was checked by LC-MS/MS analysis to ensure proper labelling of all TMT channels, excess reagent was quenched using 5% hydroxylamine (v/v) (Thermo Scientific) for 15 min at RT. After incubation, the labelled peptides were mixed 1:1 and the pooled TMT samples were acidified with 2% Formic acid (FA) and vortexed to pellet SDC. The samples were centrifuged at 20,000 g for 15 min at RT and the supernatant was transferred to a new tube and dried by vacuum centrifugation.

Enrichment of phosphorylated peptides

The TMT labelled peptide mixture was dissolved in a solution of 80% acetonitrile (ACN), 5% trifluoroacetic acid (TFA), and 1 M glycolic acid (Sigma), and incubated with 0.6 mg TiO_2 beads (Titansphere 10 µm, GL Sciences) per 100 µg peptide for 15 min at RT with vigorous shaking to enrich the phosphorylated peptides. The beads were centrifuged briefly, and the supernatant transferred to a new tube with 0.3 mg TiO_2 beads per 100 µg peptide. After 10 min incubation at RT with vigorous shaking and a brief centrifugation the supernatant was collected. The beads were subsequently washed with 80% ACN/1% TFA and 10% ACN/0.1% TFA. The supernatant with the unbound TiO_2 fraction and the washing fractions, both containing the non-modified peptides, were combined. The phosphorylated peptides were eluted from the beads by incubation with 1.5% ammonium hydroxide solution (Sigma) at RT and pH 11.3with vigorous shaking. The beads were spun down and the supernatant was passed through C8 material from a 3M Empore™ disk (Sigma). Any remaining peptides were eluted from the disk with 30% ACN and all peptide samples were dried. Since sialylated glycopeptides also bind to the TiO_2 beads(Larsen et al., 2007), the sample was deglycosylated with N-glycosidase F (Biolabs) and Sialidase A (Prozyme) in 50 mM TEAB, pH7.5 at 37°C ON.

High-pH fractionation

To reduce the complexity of the samples, non-modified and phosphophospho-peptides were fractionated by High-pH chromatography prior to nanoLC-MS/MS analysis.
The peptide samples were dissolved in 30 µL solvent A (20 mM ammonium formate, pH 9.5) and loaded onto an Acquity UPLC™M-Class CSH™C18 column (Waters) using a Dionex Ultimate 3000 HPLC system (Thermo Scientific). Approximately 100 µg of the non-modified peptide samples was fractionated, whereas the whole of the phosphopeptide samples were fractionated.
Separation of the peptides was performed using a 70-minute gradient from 2 to 95% solvent B (80% ACN, 20% solvent A) in solvent A, at a flow rate of 0.1 µL/min. The fractions were collected every 60 seconds into a final of 12 concatenated fractions in a 96-well plate (Axygen), dried by vacuum centrifugation, and stored at -20°C.

Reversed-phase nanoLC-ESI-MS/MS

Each high pH fraction was resuspended by adding 3 µL of solvent A (0.1% FA) and loaded in a volume of 2.5 µL onto an analytical column on an EASY-nLC 1000 system (Thermo Scientific).
The analytical column was a 21 cm long fused silica capillary (75 µm inner diameter) and packed with ReproSil-Pur C18 AQ 1.9 µm reversed-phase material (both resins Dr. Maisch Ammerbuch-Entringen). The peptides are eluted with an increasing concentration of organic solvent (solvent B: 95% ACN, 0.1% FA) over a gradient of 120 min in the following manner: from 2% to 25% solvent B in 100 min, 25-40% in 20 min and 40-95% in 1 min. The flow was 300 nL/min. The nLC was online connected to an Orbitrap Eclipse™ Tribrid™ mass spectrometer (Thermo Scientific) operated at positive ion mode with data-dependent acquisition. The Orbitrap acquired the full MS scan with an automatic gain control (AGC) target value of 300% ($3x10^6$) ions and a maximum injection time of 50 ms. Each MS scan is acquired at high-resolution (120,000 full width half maximum (FWHM)) at m/z 200 in the Orbitrap, with a mass range of 350-1600 Da. For the non-modified peptides, the peptide fragmentation was performed using the SPS-MS3 method with real time database searching(Schweppe et al., 2020). Briefly, each peptide was selected (0.7Da window) and fragmented in the linear ion-trap using CID with a normalized collision energy of 35% and an activation time of 10 ms. Each MSMS spectrum was subjected to a brief 20-30 ms database search against a Human uniprot reviewed database using a database search program built into the MS computer. If the resulting MSMS database search received a confident match in the database, the same ion was reselected and fragmented in the linear ion-trap. Subsequently, the 10 most intense fragment ions originating from the identified peptide were reselected, fragmented using HCD fragmentation (NCE 55), and scanned out in the orbitrap with 30.000 in resolution optimised for resolving of the TMT reporter ions. The eclipse workflow was set to automatic calculation of number of peaks that could be selected within a 3 second duty cycle. For the phosphopeptides, the fragmentation was performed using HCD NCE 36, with the following settings for ion detection; resolution 50K FWHM, maximum injection time 200 ms, and AGC target 200%. The MSMS was performed with a cycle time of 3 sec. All raw data were viewed in Thermo Xcalibur v3.0.

Mass spectrometry data analysis

The raw data were processed using Proteome Discoverer (v2.5, ThermoFisher, PD2.5) and all data were cross referenced against a Human Uniport Reviewed database. The non-modified peptides were searched for using the SEQUEST HT search algorithm only, while the phosphopeptide data were searched for initially using an in-house Mascot search algorithm and then by the SEQUEST HT search algorithm. The data were searched with 10 ppm accuracy in MS and 0.8 Da in MSMS mode (linear ion trap MSMS) for the non-modified peptides, and 0.05 Da for the phospho-peptides. The quantitation was performed using the MS3 HCD spectra for the non-modified peptides, and MS2 HCD spectra for the phospho-peptides. Database searches were performed with the following parameters: TMTpro 16-plex (Lys and N-terminal) as fixed modifications and a maximum of 2 missed cleavages for trypsin. Additionally, for the phospho-peptides, the search was performed with

phosphorylation of serine/threonine/tyrosine (S/T/Y) and deamidation of asparagine (N) as variable modifications. All identified peptides were filtered against a Decoy database using Percolator with a false discovery rate (FDR) of 0.01 (FDR < 0.01). Only peptides with rank 1 were considered for further analysis. Only proteins with more than 1 unique peptide were considered for further analysis in the non-modified group.

Quantitative analysis was based on 4 biological replicates. Quantification across the 2 sets of TMTpro 16-plex was normalized based on a common reference channel containing a mix of all samples. The relative abundances of the non-modified and phosphopeptides were normalized using PD2.5. Principal component analysis was performed in PD2.5 and Perseus v1.5.4.1 to evaluate the separation between the replicates and the sample groups. Heatmaps were prepared in Perseus v1.5.4.1 with k-means clustering and Euclidean distance. Statistical testing to identify significant differences between the noncompetent and the competent cell lines was performed using PolyStest (http://computproteomics.bmb.sdu.dk:443/app_direct/PolySTest)(Schwämmle et al., 2020) applying the Limma test with FDR<0.05. Results were evaluated using Panther (https://pantherdb.org) against the background of all human genes with FDR<0.01 cut-off to identify enriched GO annotations. Further Cluster analysis was performed using Variance sensitive fuzzy clustering (VSClust) (http://computproteomics.bmb.sdu.dk:8192/app/VSClust)(Schwämmle & Jensen, 2018). Results from the cluster analysis were further evaluated using Panther against the background of all detected peptides with FDR<0.01 cut-off. Enrichment bar plots were generated using a custom python script.

Immunoblotting

Cells on culture plates were washed twice in ice-cold PBS and lysed with modified RIPA buffer (mRIPA: 1% Triton-X, 0.1% SDS, 150 mM NaCl, 50 mM Tris pH 7.4, 2 mM EDTA, 12 mM sodium deoxycholate) freshly supplemented immediately with protease (Thermo Fisher, 78430) and phosphatase (Sigma-Aldrich, 4906845001) inhibitors. The protein concentration of the samples was measured using the Quick Start Bradford Dye Reagent (Bio-Rad, 5000205). 10 µg of total protein per sample was resolved by SDS-PAGE (4-20% gels) and transferred to Amersham Hybond P 0.45 PVDF blotting membranes (GE Healthcare, 10600023). Membranes were blocked overnight at 4°C in 5% skim milk powder or in 5% BSA and 0.1% Tween in PBS for working with phospho-specific antibodies. They were then incubated with primary antibodies overnight at 4 °C in 5% skim milk powder or in 5% BSA and 0.1% Tween in PBS. HRP-conjugated goat anti-rabbit (Dako, P0448, 1:3000), Dylight800 4xPEG conjugated anti-mouse (Cell Signalling Technology 5257P, 1:3000) or Dylight800 4xPEG conjugated anti-rabbit (Cell Signalling Technology 5151P, 1:3000) secondary antibodies were incubated for 1 hr at room temperature. The blots were developed with ECL Prime enhanced chemiluminescent detection reagent (GE Healthcare, RPN2232) and imaged using a Chemidoc MP system (BioRad), or imaged directly on the same system. Images were quantified in FIJI Version 2.16.0/1.54p and the final results are presented as a ratio of pFAK/FAK signal.

Supplemental references

Heissel, S., Bunkenborg, J., Kristiansen, M. P., Holmbjerg, A. F., Grimstrup, M., Mørtz, E., Kofoed, T., & Højrup, P. (2018). Evaluation of spectral libraries and sample preparation for DIA-LC-MS analysis of host cell proteins: A case study of a bacterially expressed recombinant biopharmaceutical protein. *Protein Expression and Purification*, *147*, 69–77. https://doi.org/10.1016/j.pep.2018.03.002

Larsen, M. R., Jensen, S. S., Jakobsen, L. A., & Heegaard, N. H. H. (2007). Exploring the Sialiome Using Titanium Dioxide Chromatography and Mass Spectrometry. *Molecular & Cellular Proteomics*, *6*(10), 1778–1787. https://doi.org/10.1074/mcp.M700086-MCP200

Schwämmle, V., Hagensen, C. E., Rogowska-Wrzesinska, A., & Jensen, O. N. (2020). PolySTest: Robust Statistical Testing of Proteomics Data with Missing Values Improves Detection of Biologically Relevant Features. *Molecular & Cellular Proteomics*, *19*(8), 1396–1408. https://doi.org/10.1074/mcp.RA119.001777

Schwämmle, V., & Jensen, O. N. (2018). VSClust: Feature-based variance-sensitive clustering of omics data. *Bioinformatics*, *34*(17), 2965–2972. https://doi.org/10.1093/bioinformatics/bty224

Schweppe, D. K., Eng, J. K., Yu, Q., Bailey, D., Rad, R., Navarrete-Perea, J., Huttlin, E. L., Erickson, B. K., Paulo, J. A., & Gygi, S. P. (2020). Full-Featured, Real-Time Database Searching Platform Enables Fast and Accurate Multiplexed Quantitative Proteomics. *Journal of Proteome Research*, *19*(5), 2026–2034. https://doi.org/10.1021/acs.jproteome.9b00860
